# Genome-wide identification of wheat (*Triticum aestivum*) expansins and expansin expression analysis in cold-tolerant and cold-sensitive wheat cultivars

**DOI:** 10.1371/journal.pone.0195138

**Published:** 2018-03-29

**Authors:** Jun-Feng Zhang, Yong-Qing Xu, Jia-Min Dong, Li-Na Peng, Xu Feng, Xu Wang, Fei Li, Yu Miao, Shu-Kuan Yao, Qiao-Qin Zhao, Shan-Shan Feng, Bao-Zhong Hu, Feng-Lan Li

**Affiliations:** 1 College of Life Science, Northeast Agricultural University, Harbin, Heilongjiang, China; 2 Harbin University, Harbin, Heilongjiang, China; Saint Mary’s University, CANADA

## Abstract

Plant expansins are proteins involved in cell wall loosening, plant growth, and development, as well as in response to plant diseases and other stresses. In this study, we identified 128 expansin coding sequences from the wheat (*Triticum aestivum*) genome. These sequences belong to 45 homoeologous copies of *TaEXPs*, including 26 *TaEXPAs*, 15 *TaEXPBs* and four *TaEXLAs*. No *TaEXLB* was identified. Gene expression and sub-expression profiles revealed that most of the *TaEXPs* were expressed either only in root tissues or in multiple organs. Real-time qPCR analysis showed that many *TaEXPs* were differentially expressed in four different tissues of the two wheat cultivars—the cold-sensitive ‘Chinese Spring (CS)’ and the cold-tolerant ‘Dongnongdongmai 1 (D1)’ cultivars. Our results suggest that the differential expression of *TaEXPs* could be related to low-temperature tolerance or sensitivity of different wheat cultivars. Our study expands our knowledge on wheat expansins and sheds new light on the functions of expansins in plant development and stress response.

## Introduction

Plant expansins are a family of non-enzymatic proteins present in the plant cell wall. They play important roles in cell wall modification, which is critical for cell growth and other biological processes [1]. They bind to glucan-coated cellulose in the cell wall, causing a reversible disruption of hydrogen bonds between cellulose microfibrils and the glucan matrix to loosen the cell wall [2, 3]. Expansins also participate in the processes of seed germination [4] and the creation of yield [5], root growth and development [6–8], stem growth [9] and internode elongation [10]. Expansins are also involved in leaf initiation and expansion [11], flowering and determining flower size [12], pollen germination and fertilization [13], and fruit growth and/or ripening [14, 15]. They were associated with nutrient-uptake efficiency [16] as well as with abiotic and biotic stress tolerance [4, 17]. Most recently, it has been reported that expansins contribute to improving germination, leaf size, fruit growth/ripening, and enhancing plants tolerance to abiotic and biotic stresses [18], suggesting that this may be a promising gene family for improving crop quality and yield.

All expansin proteins have two conserved domains, the *DPBB_1* and *Pollen_allerg_1* domains. In addition, expansins have a 20–30 amino acid signaling peptide that is important for their expression [2, 19]. Plant expansins can be divided into four subfamilies, expansins A (EXPA), expansins B (EXPB), expansin-like A (EXLA) and expansin-like B (EXLB) [20]. Individual plant species may have all or some of the four subfamilies of expansins. For example, no EXLB has been found in *Selaginella*, *Physcomitrella patens*, [21] and maize [22].

Wheat (*Triticum aestivum*) is an important grain crop worldwide and scientists finds its genome to be one of the most challenging in plants to analyze. Its hexaploid genome features three complete genomes termed A, B and D, that originated from three diploid ancestral species: *Triticum urartu* (A Genome), *Aegilops speltoides* (B Genome) and *Aegilops tauschii* (D Genome) [23]. As a result, most genes in wheat are represented by triplicate homoeologs [24–27]. Currently, only 18 *Triticum aestivum EXPs* (*TaEXPs*) have been identified, several of which are homoeologous copies of the same gene [28]. One of the 18 *TaEXPs*, the *TaEXPA1* gene, showed a similar phenotype and function in transgenic *Arabidopsis* [29].

Genome-wide analysis of expansins has been reported in *Arabidopsis* and rice [30], tobacco [31], soybean [32], tomato [33], grape [34], and maize [22]. However, no genome-wide expansin screening has been reported in wheat. In this study, we identified 128 expansin sequences from the draft sequences of the hexaploid wheat genome. *Cis*-acting element analysis revealed that many expansins are regulated by various hormones. They may be directly or indirectly involved in low-temperature stress response. We then compared the expansin expression profiles between a cold-tolerant (Dongnongdongmai 1, or D1) and a cold-sensitive (Chinese Spring, or CS) wheat cultivar. Our results suggest the possible involvement of wheat expansin in the low temperature stress response of wheat. Our study expands our knowledge of wheat expansins and provides new insights into the functions of expansins in plant development and stress response.

## Materials and methods

### Identification and sequence analysis of the wheat expansin genes

The sequences of the 18 identified previously *TaEXP* genes [28] were used to identify new *TaEXPs* using a blastn search against the *Triticum aestivum* genome sequences from GRAMENE (http://ensembl.gramene.org/) [35]. Sequences of *Triticum urartu* expansin genes [36] were also used to further identify *TaEXPs* in *Triticum aestivum* by BLAST against the IWGSC database. In addition, the expansin protein family Expan_Lol_pI (IPR007118) (https://www.ebi.ac.uk/interpro) was used to further search for expansin genes in the UniProt database (http://www.uniprot.org/). A total of 263 protein sequences were found and sequences that did not originate from the wheat genome were removed. The expansin genes from different databases were integrated and unified using the wheat IWGSC1+popseq.30 genome assembly.

The two essential structures of expansin genes, the *DPBB_1* and *Pollen_allerg_1* domains, were identified by the Conserved Domains tool (http://www.ncbi.nlm.nih.gov/Structure/cdd/wrpsb.cgi). In plants, the p12/PNP/kiwellin proteins had a *DPBB_1* domain only, and Grp2 pollen allergens proteins had a *Pollen_allerg_1* domain only. Therefore, shorter sequences that had only one of the two domains were removed from further analysis to avoid false positive results [37]. Homoeologous copies were identified by the polyploid theory service in GRAMENE. The homoeologous expansin genes that were not present in all three subgenomes (A, B and D) were considered to be incomplete because of the incomplete genome sequences of wheat (Cheuk and Houde, 2016). The expansin gene library was regarded as the main data source. The TGACv1 database (http://www.gramene.org/) was initially used as supplementary data. Sequences from *Triticum urartu* (release no. 30) and *Aegilops tauschii* (release no. 30) were deemed as a second set of supplementary data [38]. The supplemental sequences were identified by the ncbi-blast-2.2.31+ program (ftp://ftp.ncbi.nlm.nih.gov/blast/executables/blast+/2.2.31-last-win32-release/) from the main data by blast against the first and second supplementary data with the following parameters: Identity ≥ 93%, overlap_coverage ≥ 75% [39], overlap_length ≥ 300bp and gene_length ≥ 600 bp [2].

The gene sequences, including genomic sequences, transcript sequences, coding domain sequences (CDS), and promoter sequences, were downloaded from the Phytozome (https://phytozome.jgi.doe.gov/pz/portal.html) and GRAMENE. All gene IDs were unified into TGACv1 gene ID. Protein sequences were predicted using the translation tool (http://web.expasy.org/translate/). The molecular weight, isoelectric point (pI), and the grand average of hydrophobicity (GRAVY) of each *TaEXPs* were calculated by the Protparam tool (http://web.expasy.org/protparam/). The signal peptides were predicted online with SignalP 4.1 Server (http://www.cbs.dtu.dk/services/SignalP/).

The *TaEXPs* proteins were aligned using Clustal X1.83 with the default parameters except the gap opening, which was changed to 4.0 [40]. A phylogenetic tree was constructed using the neighbor-joining method in MEGA 5.0 [41] with default parameters. The bootstrap method was used for the test of phylogeny. The number of bootstrap replications was 500, and the model was equal to the input model. The MEME program (http://meme-suite.org/) was used to detect particular motifs of *TaEXP* proteins. The optimal motif width was 15 to 200 and maximum number of motifs was 20 [42]. The Web AUGUSTUS program (http://bioinf.uni-greifswald.de/augustus/submission.php) [43] and the GSDS 2.0 program (http://gsds.cbi.pku.edu.cn/) [44] were used to predict the exon–intron structures. Map Inspect software (http://mapinspect.software.informer.com/) was used to analyze the genic physical location on chromosomes. *Cis*-acting elements analysis in the 2 -kb upstream regions was performed with PlantCARE (http://bioinformatics.psb.ugent.be/webtools/plantcare/html/) [45, 46].

The overall expression and sub-expression of the wheat expansins were analyzed using previously developed databases. The overall expression profile (measured as transcripts per million) was calculated for each tissue by the UniGene EST profile in the US Department of Health National Center for Biotechnology Information website (https://www.ncbi.nlm.nih.gov/unigene/). The sub-expression profile (measured as reads per kilobase of exon model per million mapped reads) was calculated from the E-MTAB-4484 datasets of the Expression Atlas (https://www.ebi.ac.uk/gxa/home) [47]. These datasets were generated using the *Triticum aestivum* cultivar Chinese Spring. All datasets are publicly available at the above two websites. The profiles and *cis*-acting elements analysis of *TaEXPs* were made into heat maps by HemI 1.0 software (http://hemi.biocuckoo.org/) [48].

### Plant materials and treatments

Two cultivars of *Triticum aestivum*, CS and D1 [49], were used as the plant materials to investigate the expression profiles of expansin genes during cold-acclimation. Seeds were soaked into double-distilled water about 24 h at 25°C, and then sprouted on moistened filter paper for 12 h. The seedlings were transplanted into pots (four seedlings per pot) and then grown in a plant culture room at 15 ± 2°C under 16:8 h light:dark cycles using red LED and natural light. For expansin expression profiling at normal growth condition, tissues (root, tillering node, stem, and the third leaf) were collected when plants reached the three-leaf stage (~20 d growth at 15°C) were frozen immediately in liquid nitrogen and stored at −80°C for RNA extraction. Tissues were collected from 4 different plants from one pot and the same tissue was mixed together. Three different pots were used as 3 independent experiments. Results were average of these 3 independent experiments. The plants underwent low-temperature treatment when they reached the three-leaf stage. A total of 30 pots (4 plants per pot) from each cultivar were used, half of which were randomly selected and moved to a 4°C growth chamber for 0, 3, 6, 12 and 24 h. The other half were grown in the original culture room as controls. Four different tissues (root, tillering node, stem, and the third leaf) were collected at the three-leaf stage from both 4°C plants and normal temperature plants. Samples were frozen immediately in liquid nitrogen and stored at −80°C for RNA extraction. At each time point, tissues were collected from 4 different plants in one pot and the same tissue was mixed together. Three different pots were used as 3 independent experiments. Results were average of these three independent experiments. The differential *TaEXP* expression at 4°C was compared against the expression at normal temperature of the same time point (control).

### RNA isolation and real-time quantitative -PCR analysis

Total RNA was isolated from each plant tissue using a TransZol Up Plus RNA Kit (Transgen Biotech, Beijing, China), and treated with DNase I (Transgen). The quality of RNA was examined by agarose gel electrophoresis. The RNA samples were reverse transcribed to cDNAs with TransScript^®^ One-Step gDNA Removal and cDNA Synthesis SuperMix (Transgen). The cDNA was diluted five times before being used in downstream experiments.

Due to the high similarity of the homoeologous copies of each gene, the coding domain sequence (CDS) regions of the 45 *TaEXPs* were used to design for specific primers using Primer 5.0 program and Primique software (http://cgi-www.daimi.au.dk/cgi-chili/primique/front.py) [50]. Primers were then verified by blasting against genomic sequences in GRAMENE and in EST and NR/NT databases. The β-actin (Traes_1AL_E195290EF.2; Traes_1BL_D0A3F2067.1; Traes_1DL_727F09EF8.1) was used as the reference gene. All primers were listed in Supplemental S1 Table.

The Linegene 9600 fluorescence quantitative PCR detection system (Bioer Technology Co., Inc., Hangzhou, China) was used for real-time PCR experiments with the TransStart Top Green qPCR SuperMix (Transgen) in a total 20 μl reaction system, using SYBR as a reporter dye. Thermal cycling for RT-qPCR was as follows: 95°C for 20 s, 40 cycles of 95°C for 15 s, and 60°C for 20 s with luminescent reading. Melting curves were mapped by increasing the final temperature from 60 to 95°C. All the data were analyzed using the 2 ^−ΔΔCT^ method [51].

### Statistical analysis

Microsoft Excel was used for statistical analysis (Student’s t-tests). Values are presented here as means of three biological replicates; error bars indicate standard derivation (SD). Significant differences are denoted with “*” (*P* < 0.05) or “**” (*P* < 0.01).

## Results

### Identification of expansin sequences in wheat

A total of 128 expansin sequences were identified and these were named according to standard principles for consistency [20]. Sequences of the 128 expansin genes were deposited in the GRAMENE database (http://ensembl.gramene.org/) and can be accessed by the gene IDs listed in Table 1. *TaEXPA1*-*11*, *TaEXPB1*-*11* and *TaEXPB23* had been named previously [28, 52–54], so the newly identified expansins were named as *TaEXPA12* to –*29*, *TaEXPB12* to –*24* except for two that were named *TaEXPB23* and *TaEXLA1* to –*4* (Table 1). Fifty-five of the identified expansin genes were confirmed to be located on 16 chromosomes (S1 Fig), but the chromosomal locations of other genes were not determined because some genome sequences were incomplete. Majority of the expansin genes had three copies, one each from chromosomes A, B, or D. *TaEXPA3* and *TaEXPA22* had two extra copies from B and D. *TaEXPA25*, *TaEXPA26*, *TaEXPA28*, *TaEXPA29*, *TaEXPB20*, and *TaEXLA4* were not detected in chromosome A; *TaEXPA26*, *TaEXPB17* and *TaEXLA4* were not in chromosome B and *TaEXPA28* and *TaEXPA29* were not in chromosome D.

**Table 1:** Wheat expansin genes identified in this study.

Gene ID	Name	Chromo-some	Protien length	Mw(Da)	pI	GRAVY	SP	DPBB_1 domain	Pollen_allerg_1 domain
TRIAE_CS42_3AL_TGACv1_194727_AA0638220	*TaEXPA3-A*	3A	251	26231.3	6.78	-0.004	1–24	64–149	162–233
TRIAE_CS42_3B_TGACv1_223925_AA0789720	*TaEXPA3-B1*	3B	251	26248.2	6.78	-0.018	1–24	64–149	162–233
TRIAE_CS42_3B_TGACv1_222333_AA0762340	*TaEXPA3-B2*	3B	251	26232.2	6.78	-0.024	1–24	64–149	162–233
TRIAE_CS42_3DL_TGACv1_250798_AA0873340	*TaEXPA3-D1*	3D	251	26270.3	6.78	-0.006	1–24	64–149	162–233
TRIAE_CS42_3DL_TGACv1_253727_AA0895440	*TaEXPA3-D2*	3D	251	26226.3	6.78	0.006	1–24	64–149	162–233
TRIAE_CS42_3AL_TGACv1_199377_AA0669710	*TaEXPA4-A*	3A	249	27113.4	7.48	-0.122	1–22	59–146	159–230
TRIAE_CS42_3B_TGACv1_223005_AA0774980	*TaEXPA4-B*	3B	249	27035.2	6.52	-0.143	1–22	59–146	159–230
TRIAE_CS42_3DL_TGACv1_250306_AA0865790	*TaEXPA4-D*	3D	249	26914.1	6.52	-0.12	1–22	59–146	159–230
TRIAE_CS42_3AS_TGACv1_211321_AA0688560	*TaEXPA5-A*	3A	252	26769.3	9.23	-0.088	1–22	60–148	161–232
TRIAE_CS42_3B_TGACv1_224402_AA0796400	*TaEXPA5-B*	3B	257	27391.1	9.17	-0.089	1–22	66–153	166–237
F775_32564	*TaEXPA5-D*		277	29527.6	9.27	-0.075	1–22	60–147	160–231
TRIAE_CS42_4AS_TGACv1_306950_AA1015420	*TaEXPA6-A*	4A	254	27799.5	7.46	0.031	1–22	63–149	162–233
TRIAE_CS42_4BL_TGACv1_320333_AA1035610	*TaEXPA6-B*	4B	254	27718.4	7.48	0.036	1–24	63–149	162–233
TRIAE_CS42_4DL_TGACv1_344422_AA1147040	*TaEXPA6-D*	4D	254	27746.4	7.48	0.039	1–24	63–149	162–233
TRIAE_CS42_2AL_TGACv1_095133_AA0307290	*TaEXPA7-A*	2A	258	27670.7	8.09	0.072	1–19	68–154	167–234
TRIAE_CS42_2BL_TGACv1_129425_AA0383180	*TaEXPA7-B*	2B	258	27670.7	8.09	0.071	1–19	68–154	167–234
TRIAE_CS42_2DL_TGACv1_161611_AA0559500	*TaEXPA7-D*	2D	258	27684.7	8.09	0.068	1–19	68–154	167–234
TRIAE_CS42_3AS_TGACv1_212410_AA0700630	*TaEXPA8-A*	3A	246	25486.6	7.55	0.107	1–20	60–144	157–228
TRIAE_CS42_3B_TGACv1_224406_AA0796470	*TaEXPA8-B*	3B	246	25520.6	7.54	0.081	1–20	60–144	157–228
TRIAE_CS42_3DS_TGACv1_272528_AA0921790	*TaEXPA8-D*	3D	246	25460.5	7.54	0.08	1–20	60–144	157–228
TRIAE_CS42_5AL_TGACv1_374454_AA1200520	*TaEXPA9-A*	5A	268	28813.8	9.4	-0.048	1–29	76–164	177–248
TRIAE_CS42_5BL_TGACv1_406965_AA1351930	*TaEXPA9-B*	5B	266	28546.4	9.51	-0.069	1–27	74–162	175–246
TRIAE_CS42_5DL_TGACv1_435145_AA1446640	*TaEXPA9-D*	5D	266	28589.5	9.61	-0.039	1–27	74–162	175–246
TRIAE_CS42_4AS_TGACv1_307970_AA1024860	*TaEXPA12-A*	4A	259	27673.7	9.05	-0.012	1–24	66–155	168–240
TRIAE_CS42_4BL_TGACv1_321137_AA1056060	*TaEXPA12-B*	4B	282	30232.7	9.45	-0.009	1–24	66–155	168–240
TRIAE_CS42_4DL_TGACv1_342474_AA1114540	*TaEXPA12-D*	4D	259	27646.8	9.05	0.021	1–24	66–155	168–240
TRIAE_CS42_1AL_TGACv1_000407_AA0011360	*TaEXPA13-A*	1A	256	27735.5	6.78	0.094	1–26	65–151	164–235
TRIAE_CS42_5BL_TGACv1_404293_AA1294240	*TaEXPA13-B*	5B	256	27817.5	6.67	0.082	1–26	65–151	164–235
TRIAE_CS42_5DL_TGACv1_433714_AA1420290	*TaEXPA13-D*	5D	256	27811.5	6.78	0.083	1–26	65–151	164–235
TRIAE_CS42_1AL_TGACv1_000407_AA0011380	*TaEXPA14-A*	1A	254	27531.2	7.46	0.059	1–16	63–149	162–233
TRIAE_CS42_5BL_TGACv1_405270_AA1323630	*TaEXPA14-B*	5B	254	27497.1	8.05	0.065	1–16	63–149	162–233
TRIAE_CS42_5DL_TGACv1_433714_AA1420300	*TaEXPA14-D*	5D	255	27699.5	8.05	0.071	1–17	64–150	171–250
TRIAE_CS42_4AS_TGACv1_309318_AA1030760	*TaEXPA15-A*	4A	258	27651.7	8.96	-0.032	1–24	65–154	167–239
TRIAE_CS42_4BL_TGACv1_320437_AA1039060	*TaEXPA15-B*	4B	259	27489.5	8.79	0.084	1–24	66–155	168–240
F775_12787	*TaEXPA15-D*		259	27503.5	9.01	0.048	1–24	66–155	168–240
TRIAE_CS42_4AS_TGACv1_308797_AA1029560	*TaEXPA16-A*	4A	257	27081.2	9.11	-0.144	1–29	66–153	166–237
TRIAE_CS42_4BL_TGACv1_321802_AA1065630	*TaEXPA16-B*	4B	257	27217.5	8.99	-0.149	1–29	66–153	166–237
TRIAE_CS42_4DL_TGACv1_343034_AA1128010	*TaEXPA16-D*	4D	257	27108.3	9.01	-0.145	1–29	66–153	166–237
TRIAE_CS42_3AS_TGACv1_210583_AA0675440	*TaEXPA17-A*	3A	258	27696.8	8.73	-0.054	1–24	67–154	166–238
TRIAE_CS42_3B_TGACv1_227374_AA0823100	*TaEXPA17-B*	3B	261	28065.2	9.03	-0.091	1–24	67–156	169–241
TRIAE_CS42_3DS_TGACv1_271478_AA0899830	*TaEXPA17-D*	3D	261	28071.4	8.94	-0.051	1–24	67–156	169–241
TRIAE_CS42_1AL_TGACv1_000844_AA0020230	*TaEXPA18-A*	1A	259	27480.3	8.91	0.069	1–24	67–153	171–238
TRIAE_CS42_1BL_TGACv1_031377_AA0112820	*TaEXPA18-B*	1B	261	27682.6	8.91	0.081	1–29	69–155	173–240
TRIAE_CS42_1DL_TGACv1_062518_AA0215780	*TaEXPA18-D*	1D	259	27481.4	8.91	0.096	1–24	67–153	171–238
TRIAE_CS42_4AL_TGACv1_291883_AA0996900	*TaEXPA19-A*	4A	255	27641	8.05	-0.091	1–25	65–151	164–234
TRIAE_CS42_5BL_TGACv1_406879_AA1351000	*TaEXPA19-B*	5B	255	27632.1	8.56	-0.1	1–25	65–151	164–235
TRIAE_CS42_5DL_TGACv1_434954_AA1444020	*TaEXPA19-D*	5D	255	27581.9	8.35	-0.104	1–25	65–151	164–234
TRIAE_CS42_4AS_TGACv1_307135_AA1017450	*TaEXPA20-A*	4A	253	27107.8	8.32	0.045	1–24	61–148	161–232
TRIAE_CS42_4BL_TGACv1_320333_AA1035620	*TaEXPA20-B*	4B	253	27097.7	8.53	0.032	1–24	62–148	161–232
TRIAE_CS42_4DL_TGACv1_343034_AA1128030	*TaEXPA20-D*	4D	250	26696.2	8.32	0.062	1–24	62–145	158–229
TRIAE_CS42_5AL_TGACv1_374723_AA1207410	*TaEXPA21-A*	5A	282	29824.6	10.68	-0.602	1–24	65–140	
TRIAE_CS42_5BL_TGACv1_407229_AA1354640	*TaEXPA21-B*	5B	259	27737.6	8.96	-0.027	1–25	66–154	167–238
TRIAE_CS42_5DL_TGACv1_433521_AA1415520	*TaEXPA21-D*	5D	293	32414.9	10.24	-0.438		45–133	146–217
TRIAE_CS42_4AS_TGACv1_306914_AA1015010	*TaEXPA22-A*	4A	253	27566.2	6.24	0.091	1–24	62–148	161–232
TRIAE_CS42_4BL_TGACv1_320277_AA1033630	*TaEXPA22-BI*	4B	253	27445.1	6.24	0.083	1–24	62–148	161–232
TRIAE_CS42_4BL_TGACv1_320277_AA1033640	*TaEXPA22-B2*	4B	247	27172.6	6.38	-0.031	1–20	58–144	155–226
TRIAE_CS42_4DL_TGACv1_343032_AA1127880	*TaEXPA22-D1*	4D	253	27545.2	5.84	0.083	1–24	62–148	161–232
TRIAE_CS42_4DL_TGACv1_345231_AA1152290	*TaEXPA22-D2*	4D	253	27678.4	6.24	0.046	1–24	62–148	161–232
TRIAE_CS42_2AS_TGACv1_114363_AA0366370	*TaEXPA23-A*	2A	261	27546	8.9	0.007	1–24	69–157	170–240
TRIAE_CS42_2BS_TGACv1_146019_AA0453260	*TaEXPA23-B*	2B	262	27524.9	8.9	-0.003	1–29	70–158	171–241
TRIAE_CS42_2DS_TGACv1_177171_AA0567520	*TaEXPA23-D*	2D	261	27547	8.9	0.035	1–28	69–157	170–240
TRIAE_CS42_3AS_TGACv1_210530_AA0674760	*TaEXPA24-A*		264	28433.5	8.56	-0.076	1–23	71–160	173–245
TRAES3BF143800010CFD	*TaEXPA24-B*	3B	264	28442.4	8.06	-0.058	1–23	71–160	173–245
F775_19619	*TaEXPA24-D*		264	28386.3	8.61	-0.089	1–23	71–160	173–245
TRIAE_CS42_4BL_TGACv1_320333_AA1035630	*TaEXPA25-B*	4B	259	27424	6.87	0.043	1–22	66–154	167–237
TRIAE_CS42_4DL_TGACv1_343034_AA1128020	*TaEXPA25-D*	4D	258	27123.7	7.55	0.153	1–25	65–153	166–236
Traes_7DL_5812FED4E	*TaEXPA26-D*	7D	268	28389.3	8.98	-0.075	1–22	61–174	184–264
TRIAE_CS42_7AL_TGACv1_556942_AA1773860	*TaEXPA27-A*	7A	260	27811.6	7.48	-0.017	1–20	69–156	169–240
TRIAE_CS42_7BL_TGACv1_577039_AA1863550	*TaEXPA27-B*	7B	262	28059.9	7.48	-0.021	1–22	71–158	171–242
TRIAE_CS42_7DL_TGACv1_604808_AA2001830	*TaEXPA27-D*	7D	261	27972.8	7.46	-0.001	1–21	70–157	170–241
TRIAE_CS42_4BL_TGACv1_320437_AA1039040	*TaEXPA28-B*	4B	259	27607.8	8.7	0.116	1–24	49–165	175–255
TRIAE_CS42_4BL_TGACv1_320277_AA1033650	*TaEXPA29-B*	4B	253	27737.4	6.78	0.041	1–24	44–158	168–247
TRIAE_CS42_1AL_TGACv1_002200_AA0039680	*TaEXPB1-A*	1A	265	28714.1	4.98	-0.264	1–24	78–156	170–245
TRIAE_CS42_1BL_TGACv1_034214_AA0143800	*TaEXPB1-B*	1B	270	29277.7	4.98	-0.236	1–24	83–161	175–250
TRIAE_CS42_1DL_TGACv1_062107_AA0208850	*TaEXPB1-D*	1D	264	28687.1	4.98	-0.248	1–24	77–155	169–244
TRIAE_CS42_1AL_TGACv1_000456_AA0012510	*TaEXPB8-A*	6A	273	29962.9	9.39	-0.274	1–26	80–160	174–253
TRIAE_CS42_1BL_TGACv1_031113_AA0108080	*TaEXPB8-B*	6B	273	29736.7	9.06	-0.194	1–26	80–160	174–253
TRIAE_CS42_1DL_TGACv1_063167_AA0224540	*TaEXPB8-D*	6D	273	29890.8	9.48	-0.259	1–26	80–160	174–253
TRIAE_CS42_6AS_TGACv1_485310_AA1542890	*TaEXPB7-A*	1A	270	29137.9	6.5	-0.181	1–26	83–161	175–250
TRIAE_CS42_6BS_TGACv1_513750_AA1648570	*TaEXPB7-B*	1B	270	29219.1	6.5	-0.127	1–26	83–161	175–250
TRIAE_CS42_6DS_TGACv1_542511_AA1723160	*TaEXPB7-D*	1D	270	29203	6.93	-0.174	1–26	83–161	175–250
TRIAE_CS42_1AL_TGACv1_003247_AA0048900	*TaEXPB10-A*	1A	271	29853.7	8.81	-0.256	1–24	78–158	172–251
TRIAE_CS42_1BL_TGACv1_032979_AA0135990	*TaEXPB10-B*	1B	271	29976.9	8.81	-0.269	1–24	78–158	172–251
TRIAE_CS42_1DL_TGACv1_062861_AA0221130	*TaEXPB10-D*	1D	271	30037	8.81	-0.259	1–24	78–158	172–251
TRIAE_CS42_2AL_TGACv1_093830_AA0287660	*TaEXPB12-A*	2A	281	29498.3	9.17	-0.125	1–25	80–160	174–260
TRIAE_CS42_2BL_TGACv1_129731_AA0394090	*TaEXPB12-B*	2B	281	29494.3	9.16	-0.104	1–25	80–160	174–260
TRIAE_CS42_2DL_TGACv1_158079_AA0508190	*TaEXPB12-D*	2D	281	29496.3	9.16	-0.105	1–25	80–160	174–260
TRIAE_CS42_6AL_TGACv1_471607_AA1511690	*TaEXPB13-A*	6A	289	30322.7	9.45	-0.136	1–25	99–180	194–269
TRIAE_CS42_6BL_TGACv1_500765_AA1609400	*TaEXPB13-B*	3B	289	30349.1	9.49	0.002	1–25	99–180	194–269
TRIAE_CS42_6DL_TGACv1_527377_AA1703040	*TaEXPB13-D*	6D	289	30308.7	9.45	-0.139	1–25	99–180	194–269
TRIAE_CS42_5AS_TGACv1_393897_AA1277020	*TaEXPB14-A*	5A	323	35331.1	6.64	-0.215	1–26	108–186	200–281
TRIAE_CS42_5BS_TGACv1_423497_AA1378190	*TaEXPB14-B*	5B	341	37470.6	7.51	-0.094	1–27	107–185	199–280
TRIAE_CS42_5DS_TGACv1_457565_AA1487840	*TaEXPB14-D*	5D	347	38014.2	7.95	-0.135	1–28	113–191	205–286
TRIAE_CS42_2AL_TGACv1_094343_AA0296260	*TaEXPB15-A*	2A	265	28794.1	9.07	-0.163	1–22	78–156	171–245
TRIAE_CS42_2BL_TGACv1_131181_AA0424020	*TaEXPB15-B*	2B	265	28791.1	9.22	-0.174	1–22	78–156	171–245
TRIAE_CS42_2DL_TGACv1_159779_AA0542610	*TaEXPB15-D*	2D	265	28779	9.24	-0.217	1–23	78–156	171–245
TRIAE_CS42_6AL_TGACv1_471087_AA1502540	*TaEXPB16-A*	6A	287	29956.1	8.74	-0.114	1–26	94–176	190–267
TRIAE_CS42_6BL_TGACv1_503053_AA1627120	*TaEXPB16-B*	6B	287	30016.3	8.85	-0.083	1–26	94–176	190–267
TRIAE_CS42_6DL_TGACv1_526554_AA1687000	*TaEXPB16-D*	6D	287	30107.2	8.74	-0.135	1–26	94–176	190–267
TRIAE_CS42_6AL_TGACv1_471607_AA1511700	*TaEXPB17-A*	6A	265	27780.1	5.56	-0.099	1–27	74–155	169–245
TRIAE_CS42_6DL_TGACv1_526693_AA1690080	*TaEXPB17-D*	6D	266	27750.3	6.27	-0.042	1–27	74–156	170–246
TRIAE_CS42_2AL_TGACv1_093830_AA0287650	*TaEXPB18-A*	2A	261	27787.7	8.69	-0.269	1–27	65–145	159–240
TRIAE_CS42_2BL_TGACv1_129731_AA0394080	*TaEXPB18-B*	2B	261	27912.8	8.8	-0.271	1–27	65–145	159–240
TRIAE_CS42_2DL_TGACv1_158079_AA0508200	*TaEXPB18-D*	2D	261	27859.7	8.57	-0.281	1–27	65–145	159–240
TRIAE_CS42_1AL_TGACv1_003486_AA0050020	*TaEXPB19-A*	1A	274	30222.4	9.44	-0.362	1–25	83–161	175–254
TRIAE_CS42_1BL_TGACv1_030851_AA0102320	*TaEXPB19-B*	1B	274	30186.5	9.36	-0.303	1–25	83–161	175–254
TRIAE_CS42_1DL_TGACv1_062107_AA0208840	*TaEXPB19-D*	1D	274	30189.4	9.44	-0.339	1–25	83–161	175–254
TRIAE_CS42_3B_TGACv1_222229_AA0760180	*TaEXPB20-B*	3B	300	31366.2	5.17	-0.205	1–25	108–188	202–279
TRIAE_CS42_3DL_TGACv1_250163_AA0863360	*TaEXPB20-D*	3D	314	32644.6	5.15	-0.239	1–27	122–202	216–293
TRIAE_CS42_5AL_TGACv1_375143_AA1216890	*TaEXPB21-A*	5A	308	32331.6	5.13	0.122	1–26	73–164	178–254
TRIAE_CS42_5BL_TGACv1_404585_AA1305050	*TaEXPB21-B*	5B	309	32456.8	5.48	0.09	1–26	73–164	178–255
TRIAE_CS42_5DL_TGACv1_433716_AA1420380	*TaEXPB21-D*	5D	308	32213.5	5.43	0.119	1–26	73–164	178–254
TRIAE_CS42_2AS_TGACv1_115226_AA0371610	*TaEXPB22-A*	2A	253	26463	8.31	-0.02	1–27	65–146	160–233
TRIAE_CS42_2BS_TGACv1_146193_AA0458020	*TaEXPB22-B*	2B	252	26420.9	8.31	0	1–26	64–145	159–232
TRIAE_CS42_2DS_TGACv1_179985_AA0609770	*TaEXPB22-D*	2D	254	26741.4	7.5	0.045	1–27	65–146	160–233
TRIAE_CS42_2AL_TGACv1_093830_AA0287670	*TaEXPB24-A*	2A	262	26924.6	7.52	0.188	1–26	73–153	167–242
TRIAE_CS42_2BL_TGACv1_129731_AA0394100	*TaEXPB24-B*	2B	262	26981.6	8.05	0.121	1–26	73–153	167–242
TRIAE_CS42_2DL_TGACv1_158079_AA0508180	*TaEXPB24-D*	2D	262	26873.5	7.52	0.2	1–26	73–153	167–242
TRIAE_CS42_5AL_TGACv1_374686_AA1206430	*TaEXLA1-A*	5A	275	29628	9.5	-0.062	1–30	69–132	161–238
TRIAE_CS42_4BL_TGACv1_320494_AA1041290	*TaEXLA1-B*	4B	275	29527	9.39	-0.013	1–30	69–132	161–238
Traes_4DL_74A03B3BE	*TaEXLA1-D*	4D	277	29663.2	9.35	-0.01	1–32	71–134	163–240
TRIAE_CS42_5AL_TGACv1_377017_AA1243240	*TaEXLA2-A*	5A	273	28875	8.78	0.025	1–27	66–144	159–236
TRIAE_CS42_4BL_TGACv1_322143_AA1068920	*TaEXLA2-B*	4B	268	28615.6	8.31	-0.045	1–22	61–139	154–231
TRIAE_CS42_4DL_TGACv1_344616_AA1148420	*TaEXLA2-D*	4D	273	28934.9	7.98	-0.004	1–27	66–144	159–236
TRIUR3_10907	*TaEXLA3-A*		270	28368.8	5.61	-0.083		63–141	155–232
TRIAE_CS42_2BL_TGACv1_129773_AA0395490	*TaEXLA3-B*	2B	271	28685.5	5.75	-0.023	1–25	64–142	157–225
Traes_4DL_7453A191F	*TaEXLA3-D*	4D	271	28883.9	6.71	-0.023	1–25	64–142	156–233
Traes_4DL_C95A997A8	*TaEXLA4-D*	4D	270	28670.5	5.39	0.048	1–25	68–142	156–232

New genes were named following the known genes. Protein length, SP, and two domains were shown beside amino acids numbers. MW, molecular weight; pI, isoelectric point.

Sequence comparison in the GRAMENE database revealed that the 128 expansin sequences belong to 45 homoeologous *TaEXPs*, including 26 members of *TaEXPAs*, 15 members of *TaEXPBs* and four members of *TaEXLAs*. No EXLB sequence was identified. The 45 homoeologous *TaEXPs* were further confirmed by local BLAST analysis with the following parameters: identity ≥ 93%, overlap_coverage ≥ 75% [39], overlap_length ≥ 300 bp and gene_length ≥ 600 bp [2].

Only 11 of the 45 *TaEXPs* have been previously reported [28] and the remaining 34 were newly identified expansins in wheat. All the identified expansin sequences had two conserved essential domains, *DPBB_1* and *Pollen_allerg_1*. The predicted proteins ranged from 246 to 347 amino acids with an average of 266.64 amino acids. Their predicted molecular weights ranged from 25.46 to 38.14 kDa. The *pI* values ranged from 4.98 to 10.68. The GRAVY values ranged from −0.602 to 0.2. The number of predicted *DPBB_1* domains were 85–114 amino acids in *TaEXPAs*, 78–82 in *TaEXPBs* and 63–78 in *TaEXLAs*. The number of predicted *Pollen_allerg_1* domains were 67–79 amino acids in *TaEXPAs*, 71–81 in *TaEXPBs* and 77–78 in *TaEXLAs* (Table 1). The homoeologous *TaEXPs* were similar in their protein properties, but different *TaEXPs* were significantly different in their protein properties.

### Phylogenetic and structural analysis of the identified wheat expansins

**Fig 1:**
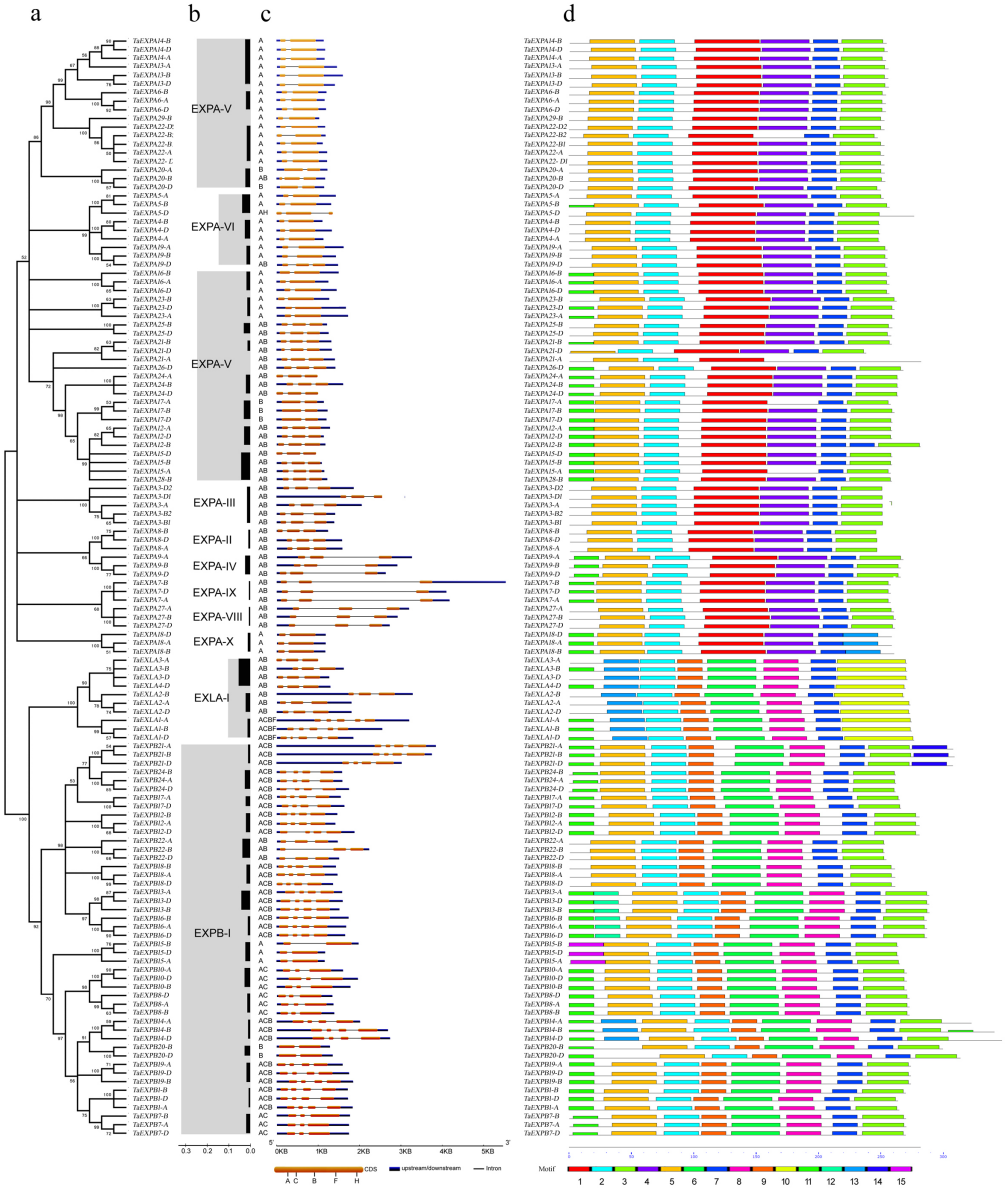
Phylogenetic analysis of wheat expansins. a. Phylogenetic tree of the 128 expansin sequences; b. Evolutionary branches of wheat expansins; c. Analysis of gene structure, coding domain sequences (CDSs), 5’/3’ untranslated region and exson/introns; d. Motif analysis of the 128 expansin sequences.

To better understand the relationships among the different expansins, the 128 predicted expansin proteins were used to establish a phylogenetic tree. This phylogenetic tree had three major branches that separate *TaEXPAs*, *TaEXPBs* and *TaEXLAs* (Fig 1a). Nine evolutionary branches were found, based on the grouping method of the *Arabidopsis* and rice expansin gene families [2] with bootstrap support values > 50% (Fig 1b, longitudinal). *TaEXPAs* had seven branches (EXPA-II, EXPA-III, EXPA-IV, EXPA-V, EXPA-VI, EXPA-VIII, and EXPA-IX), while only one branch was found in *TaEXPBs* (EXPB-I) and *TaEXLAs* (EXLA-I). The EXPA-V clade was the largest clade with 15 *TaEXPA* members. *TaEXPBs* only had the EXPB-I branch and *TaEXLAs* belonged to the EXLA-I branch. This showed that *TaEXPAs* were intricate in the phylogeny process. Two abundant EXPB-I sub-branches in wheat have been previously verified in a study related to the gramineous EXPB subfamily [3]. However, the EXPA-V branch had not been previously identified in wheat and the EXPA-IV branch was the largest clade in tobacco [31].

The phylogenetic tree showed the linearized distance that exists among *TaEXPs* and demonstrated the nature of all of their evolutionary branches (Fig 1b, horizontal). The linearity of all expansin genes was shorter than 0.1. The EXPB-I genes were longer than 3.0, the EXPA-V were 2.0 to 3.0, the EXLA-I and EXPA-VI were 1.0 to 2.0, and other genes were shorter than 0.05.

Gene structure analysis revealed the relationship among the CDS region, 5’/3’ untranslated region and exon/intron structure using GSDS software (Fig 1c). It showed that the gene region and structure of each *TaEXPs* homoeologous copy were similar. The pattern of intron insertion was classified into five categories of ACBFH [2, 30, 34]. *TaEXPAs* had one or two introns (A, B, AB or AH); *TaEXPBs* had one to three introns (A, B, AB, AC or ACB); *TaEXLAs* had two or four introns (AB and ACBF). By comparing among each of the *TaEXPs* homoeologous copies, the insertions of H, B and A were identified from *TaEXPA5-D*, *TaEXPA19-D* and *TaEXPA20-B*. Extension of A and B introns, which were longer than exons, were identified from *TaEXPA7*, *TaEXPA9*, *TaEXPA27*, *TaEXPB12-D*, *TaEXPB15-B*, and *TaEXPB22-B*. The proportion of intron extended genes was about 9.4% in wheat genome and 14.8% in maize [22], but it was 86.5% in tobacco [31], 76.3% in tomato [33] and 81.3% in soybean [32].

### Analysis of *TaEXP* protein domains and motifs

The *TaEXP* proteins contain three major domains: the signal peptide (SP), the *DPBB_1* domain, and the *Pollen_allerg_1* domain (S2 Fig). Similar to a previous study [33], the α and β insertions [55] were identified in our newly identified *TaEXPs*. The α-insertions were present in the *TaEXPAs* with seven conserved amino acids, GGWCNPP; the β-insertions were present in *TaEXPBs* and *TaEXLAs*, with only one conserved amino acid G.

A total of 15 motifs were identified (S3 Fig). Motifs 2 and 7 were present in most *TaEXPs* (Fig 1d). Motif 2 contained five specific amino acids (GCGCC) and was present in the conserved *DPBB_1* domain (pfam03330) before α and β insertions. Motif 7 also contained five specific amino acids [WM (Met) WGW] and this motif was mapped in the 3’ part of the *Pollen_allerg_1* domain (pfam01357). In *TaEXPB13* and *TaEXPB16*, Motif 7 was present in the signal peptide region. Motifs 3 and 5 were found in both *TaEXPAs* and *TaEXPBs*, while Motifs 10 and 13 were identified in *TaEXLAs*. Motifs 1 and 4 were found in *TaEXPAs*, while Motifs 6, 8 and 9 were localized in *TaEXPBs* and *TaEXLAs*. Most motifs of *TaEXPA* were conserved except *TaEXPA21-A*.

### Analysis of overall expression and subexpression of the wheat expansins

To better understand the functions of the expansins, the overall expression and subexpression of expansins were investigated using previously developed databases as described in the Materials and Methods section.

**Fig 2:**
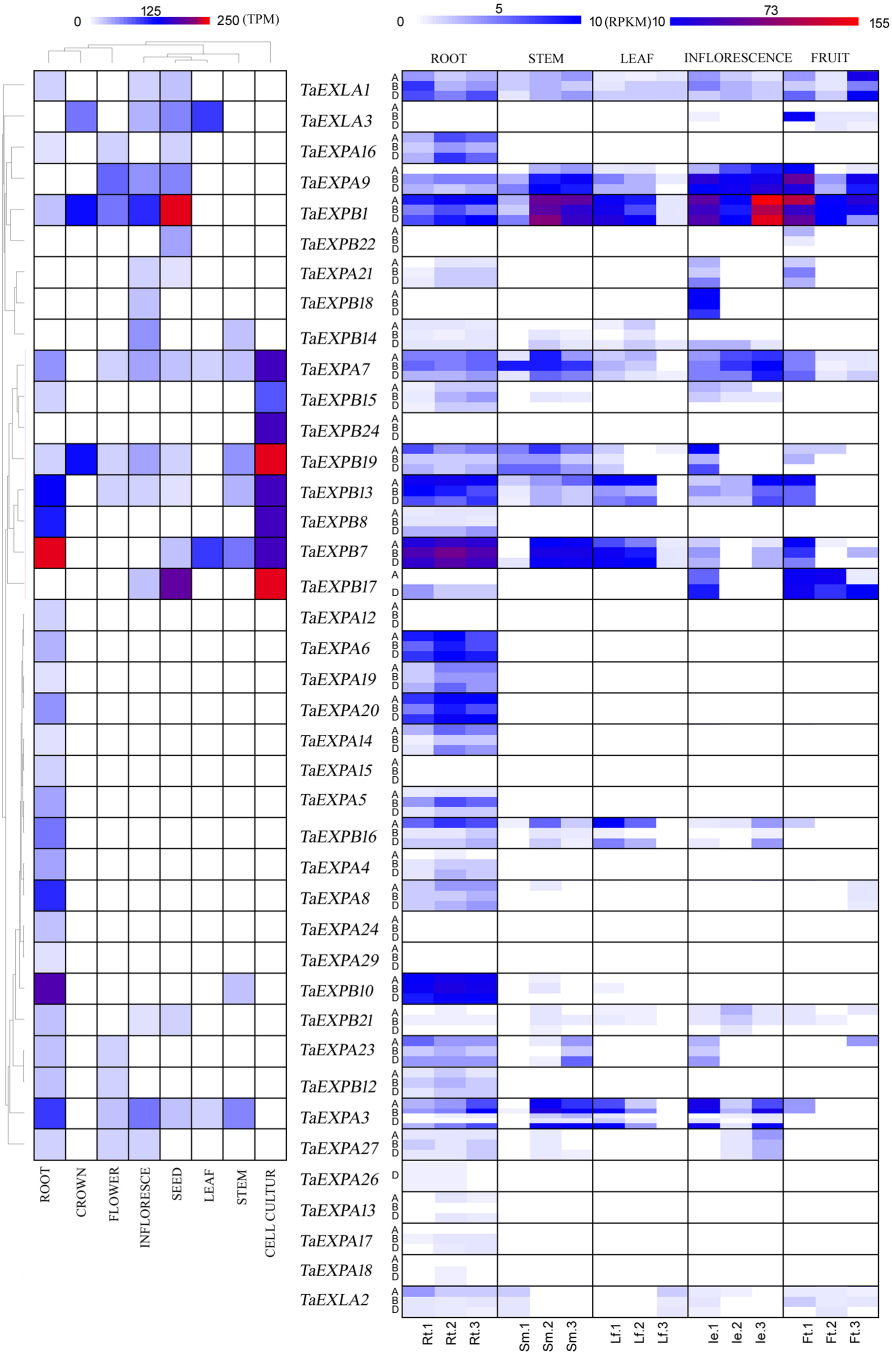
Expansin gene expression and subexpression profiles in different tissues. Expression profiles were expressed as transcripts per million. The subexpression profiles were expressed as reads per kilobase of exon model per Million mapped reads (FPKM). A, B and D represent subgenomes A, B, and D. Columns are labeled to show expansion expression as follows: Rt 1: in root during the cotyledon emergence stage, Rt 2: in root during the three leaf stage, Rt 3: in root during the maximum stem length stage; Sm 1: in stem during the half of flower open stage, Sm 2: in stem at the beginning of stem elongation, Sm 3: in stem during two nodes or internodes visible stages; Lf 1: in leaf during the main shoot and axillary shoots visible at three nodes stage, Lf 2: in stem during cotyledon emergence, Lf 3: in leaf during fruit formation; Ie 1: in inflorescence during the half of flower open stage, Ie 2: in inflorescence during the two nodes or internodes visible stage; Ie 3: in inflorescence at maximum stem length; Ft 1: in fruit during fruit formation (30 to 50% fruit formation), Ft 2: in fruit during fruit formation (70% to final size), Ft 3: in fruit during fruit ripening.

The overall expression measures the expression of organs mixed from different time points. Our results showed that 12 genes were expressed only in root tissue. *TaEXPA3*, *TaEXPA8*, *TaEXPB7*, *TaEXPB8*, *TaEXPB10*, and *TaEXPB13*, were expressed at a higher level in root than in other organs. Eighteen *TaEXPs* were expressed in some of the reproductive organs, including crown, flower, inflorescence, and seed. *TaEXPB1* and *TaEXPB19* were expressed in all these four organs. Only four genes, specifically *TaEXPA3*, *TaEXPA7*, *TaEXPB7*, and *TaEXLA3*, were expressed in leaves. Two *TaEXPAs* and five *TaEXPBs* were expressed in stems. *TaEXPA7* and seven *TaEXPBs* showed prominent expression in cell culture. *TaEXPA3*, *TaEXPA7*, *TaEXPB7*, *TaEXPB13*, and *TaEXPB19* were expressed in six of the eight tissues and/or organs (Fig 2).

For the subexpression profile, which measures the expression in different organs under the same growth conditions, our results showed that most of the homoeologous copies had different expressions, but the differences were not statistically significant. No preferred expression was observed in the three subgenomes (A, B, or D), while different expressions were observed in the same organ but different developmental stages. Overall, expansin genes were expressed broadly in roots. *TaEXPB1* had the highest expression among all the expansin genes. The expression reached its highest level at the late stage of inflorescence (le. 3 in Fig 2) and early fruit formation (ft. 1 in Fig 2). Its expression was also relatively high during early stem elongation stage (sm. 2 in Fig 2). The expressions of *TaEXPA3*, *TaEXPA7*, *TaEXPA9*, *TaEXPB7*, *TaEXPB16*, and *TaEXPB19* were significantly higher during the early stem elongation stage (sm. 2 in Fig 2), but decreased during the second half of the flower opening stage, suggesting that these genes were involved in stem elongation.

### Analysis of wheat expansin *cis*-acting elements

**Fig 3:**
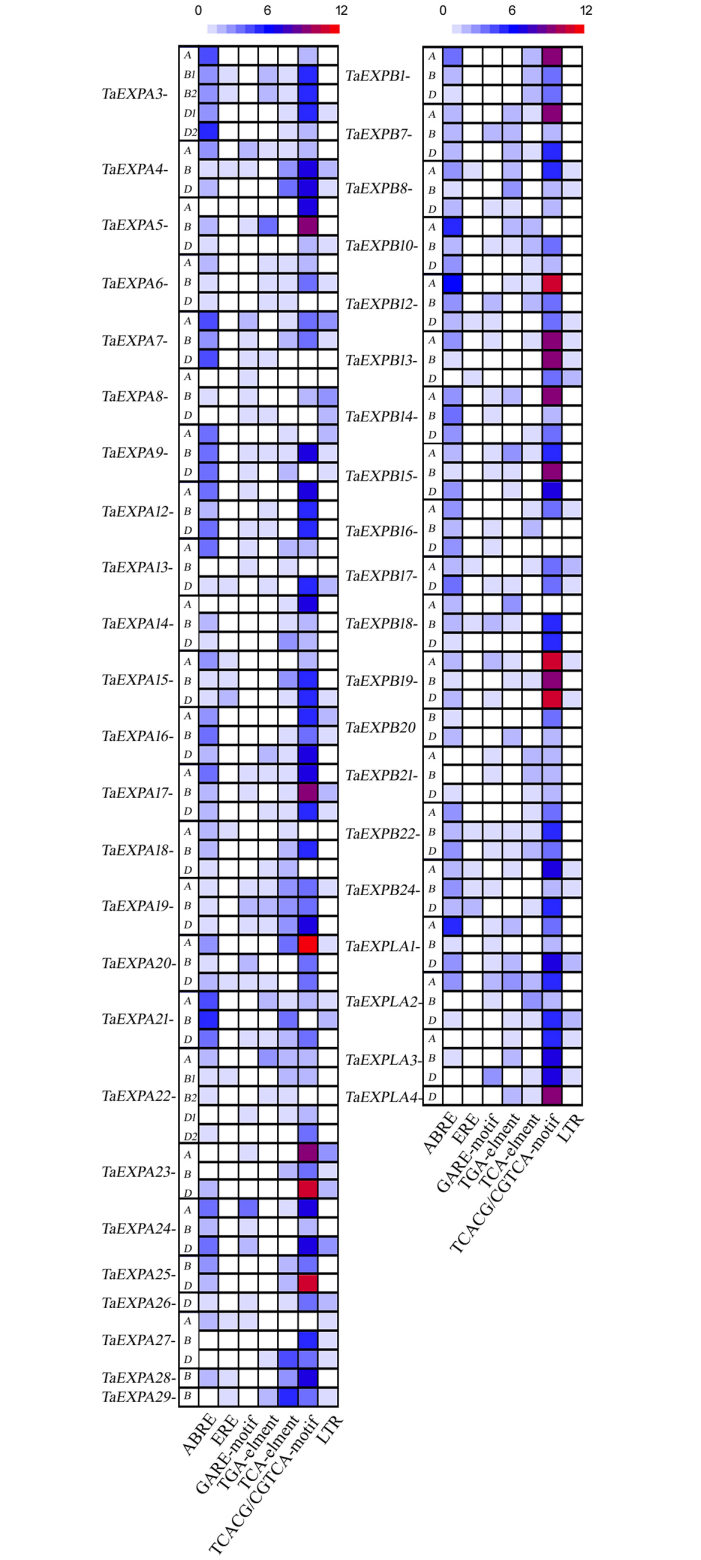
Heat map of *cis*-acting elements of expansin genes. Color indicates the number of *cis*-acting elements (0–12) in each expansin gene.

To further understand the expression and regulation of the 128 wheat expansins identified in the present study, the *cis*-acting elements, which play important roles in gene expression, were analyzed. *Cis*-acting elements are regions of non-coding DNA and are located in the promoters region. Seven *cis*-acting elements were identified (Fig 3 and S2 Table) including ABRE (*cis*-acting element involved in the abscisic acid response), ERE (ethylene-responsive element), GARE-motif (gibberellin-responsive element), TGA-element (auxin-responsive element), TCA (*cis*-acting element involved in salicylic acid response), CGTCA/TGACG-motif (*cis*-acting regulatory element involved in the MeJA-response) and LTR (*cis*-acting element involved in low-temperature response). The CGTCA/TGACG-motifs were the most abundant *cis*-acting elements, with an average of 4.9 elements per gene. The CGTCA/TGACG-motifs activate the defense mechanisms in plants in response to environmental stress, such as low temperature or drought. The second most abundant *cis*-acting element was ABRE, which is involved in the response to abscisic acid. Under severe environmental changes, such as drought, low temperature, or high salt, plants produce abscisic acid, which in turn, activates genes that respond to these stressors via the ABRE *cis*-acting element. In addition, LTR, a *cis*-acting element that is responsible for gene expression under low temperature stress, was identified in 48 of the 128 expansin genes examined (S2 Table). These results strongly suggest that some of the expansin genes might be responsible for the low-temperature stress response in wheat.

### Comparison of expansin expressions between cold-tolerant and cold-sensitive wheat cultivars

To investigate if expansins were involved in the low-temperature response of wheat, two wheat (*Triticum aestivum*) cultivars, the cold-tolerant D1 and the cold-sensitive CS cultivars, were used to compare the expression of expansin under normal and low temperatures at the three-leaf stage.

#### Phenotypical differences between CS and D1 cultivars

**Fig 4:**
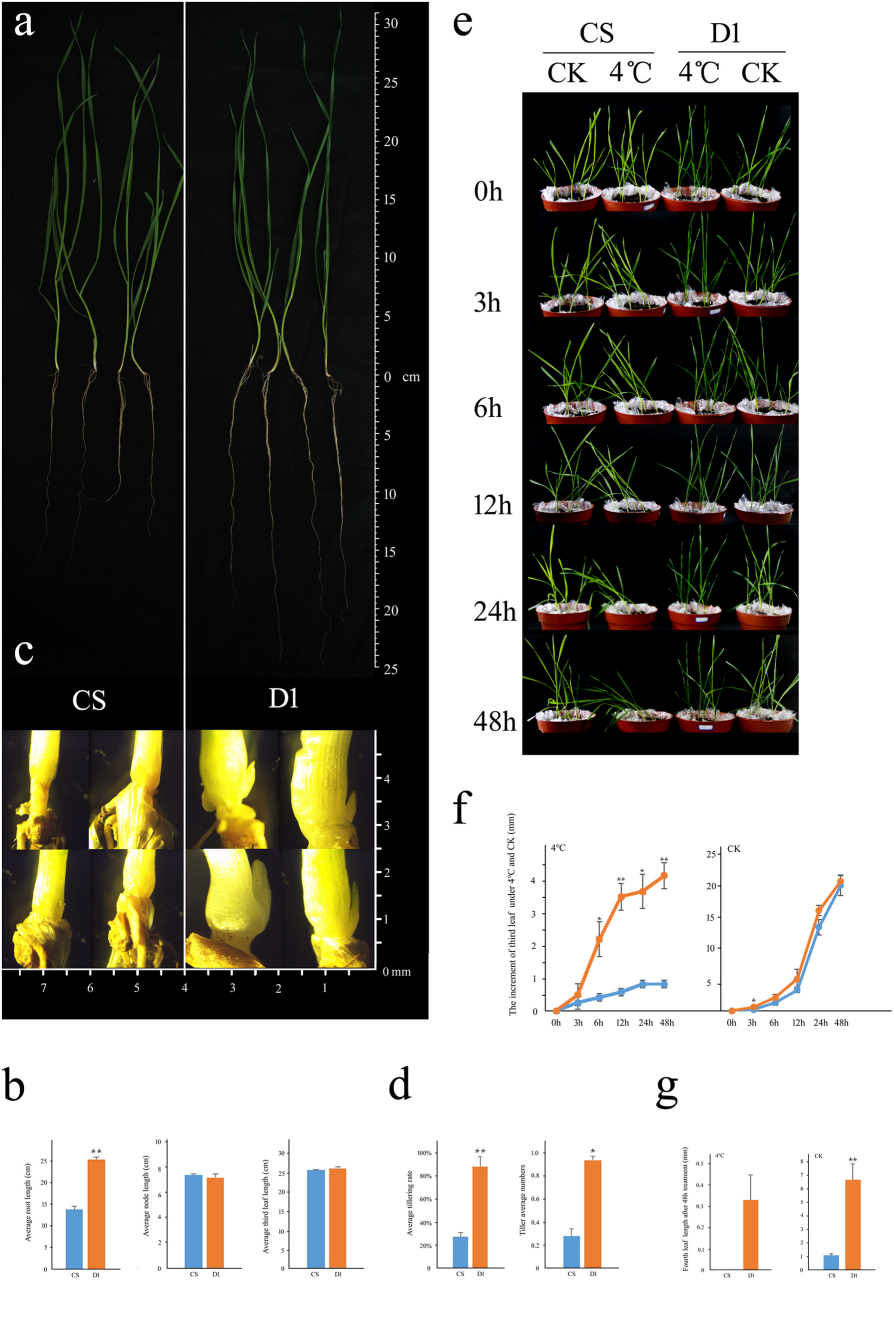
Phenotypic analysis of the wheat cultivars Chinese Spring (CS) and Dongnongdongmai 1 (D1). a. Morphology of CS and D1 plants (including roots); b. Average root length, node length and third leaf length of CS and D1, unit: cm; c. Morphology of tilling nodes of CS and D1 at the same magnification (unit: mm); d. Average tilling rate and tiller numbers of CS and D1. e. Morphology of CS and D1 plants under 4°C treatment; CK: control (growing at 15°C); f. growth of the third leaf under 4°C treatment.

Under normal growth condition (15°C), the aboveground parts of the plants were similar, but the roots of D1 were significantly longer than roots of CS plants (Fig 4a and 4b). The tillering capacity was also much higher in D1 than in CS plants (Fig 4c). Both the tillering rate and number of tillers produced in D1 plants were significantly higher than that of CS plants (Fig 4d). Under low temperature treatment (4°C), the leaves of the CS cultivar gradually collapsed while the control (CS at 15°C) and the D1 leaves grew normally (Fig 4e). The growth of the third leaf was slower for both CS and D1 plants, but the growth of D1 was significantly faster than that of the CS third leaves (*P* < 0.05; Fig 4f). After 48 h low temperature treatment, the fourth leaf emerged in D1, but was not observed in CS plants (Fig 4g).

#### *TaEXP* expression of CS and D1 cultivars under normal temperature

**Fig 5:**
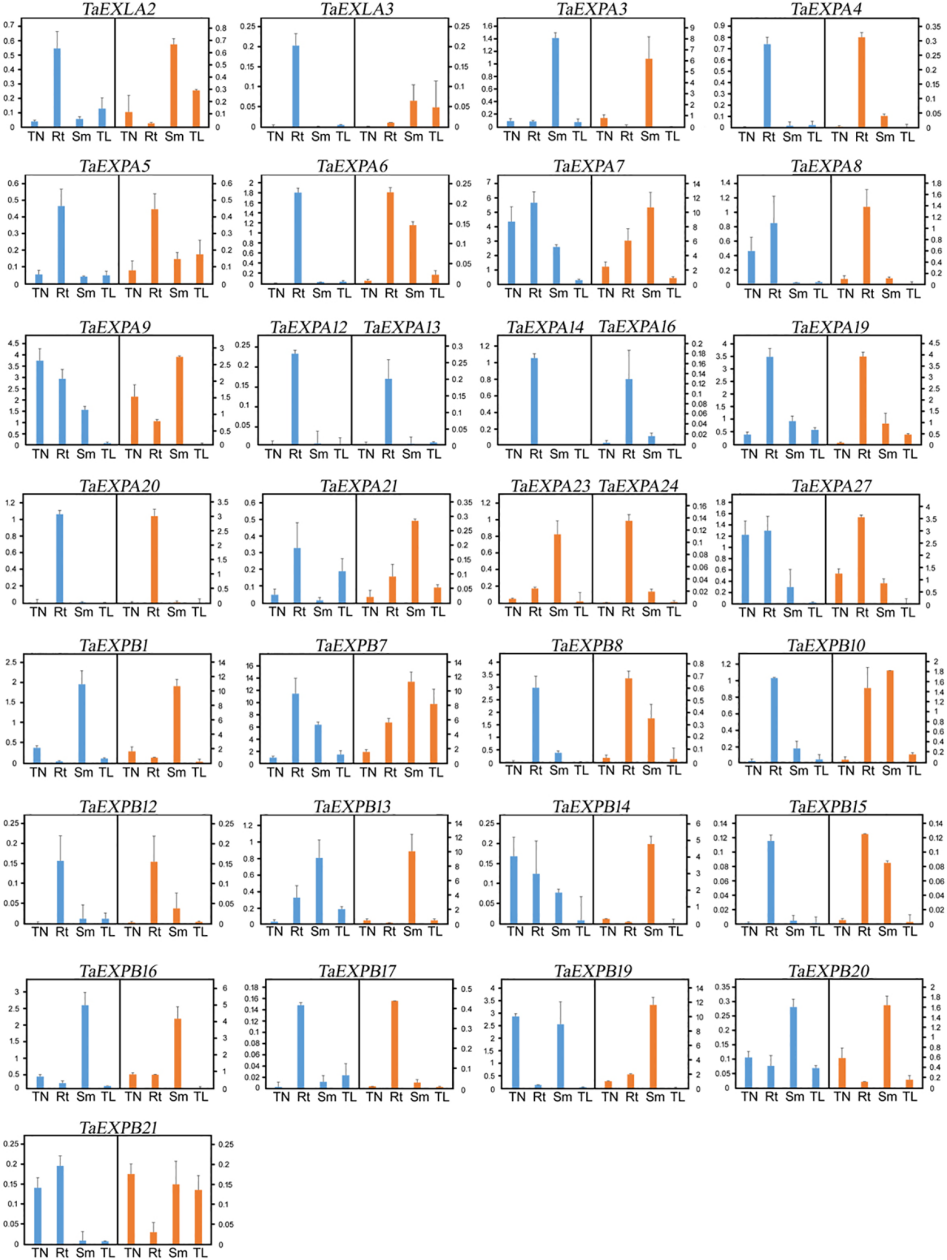
RT-qPCR of wheat expansin expressions in CS (blue) and D1 (orange) during the three leaf stage under normal growth condition. Note that certain genes (such as *TaEXPA12* and *TaEXPA13*) were only shown their expression in CS (blue) and certain other genes (such as *TaEXPA23* and *TaEXPA24*) were only shown their expression in D1 (orange). It indicates that the expression of these genes was only detected in cultivar, but not the other.

Our preliminary data showed that some of the *TaEXP* genes had a very low (< 0.1) or undetectable level of expression. After excluding these genes, we selected 32 genes to examine the expression in CS and D1 plants under normal growth conditions to receive a baseline expression profile (Fig 5). Our results showed that some of the *TaEXP* genes, such as *TaEXLA2*, *TaEXPA3*, *TaEXPA4*, *TaEXPA19*, *TaEXPB1*, and *TaEXPB12* had similar expression patterns in these two cultivars. Many other *TaEXP* genes showed either a different expression in a particular tissue (such as *TaEXLA 2* and *TaEXLA3* in roots), or different expression in all four tested tissues (such as *TaEXB21*). *TaEXPA23* and *TaEXPA24* were only expressed in D1, while *TaEXP12*, *TaEXPA13*, *TaEXP14*, and *TaEXPA16* were only expressed in CS, suggesting that these genes might be responsible for the phenotypic differences (Fig 4) between these two cultivars.

#### Differential *TaEXP* expression between CS and D1 cultivars under low-temperature treatment

**Fig 6:**
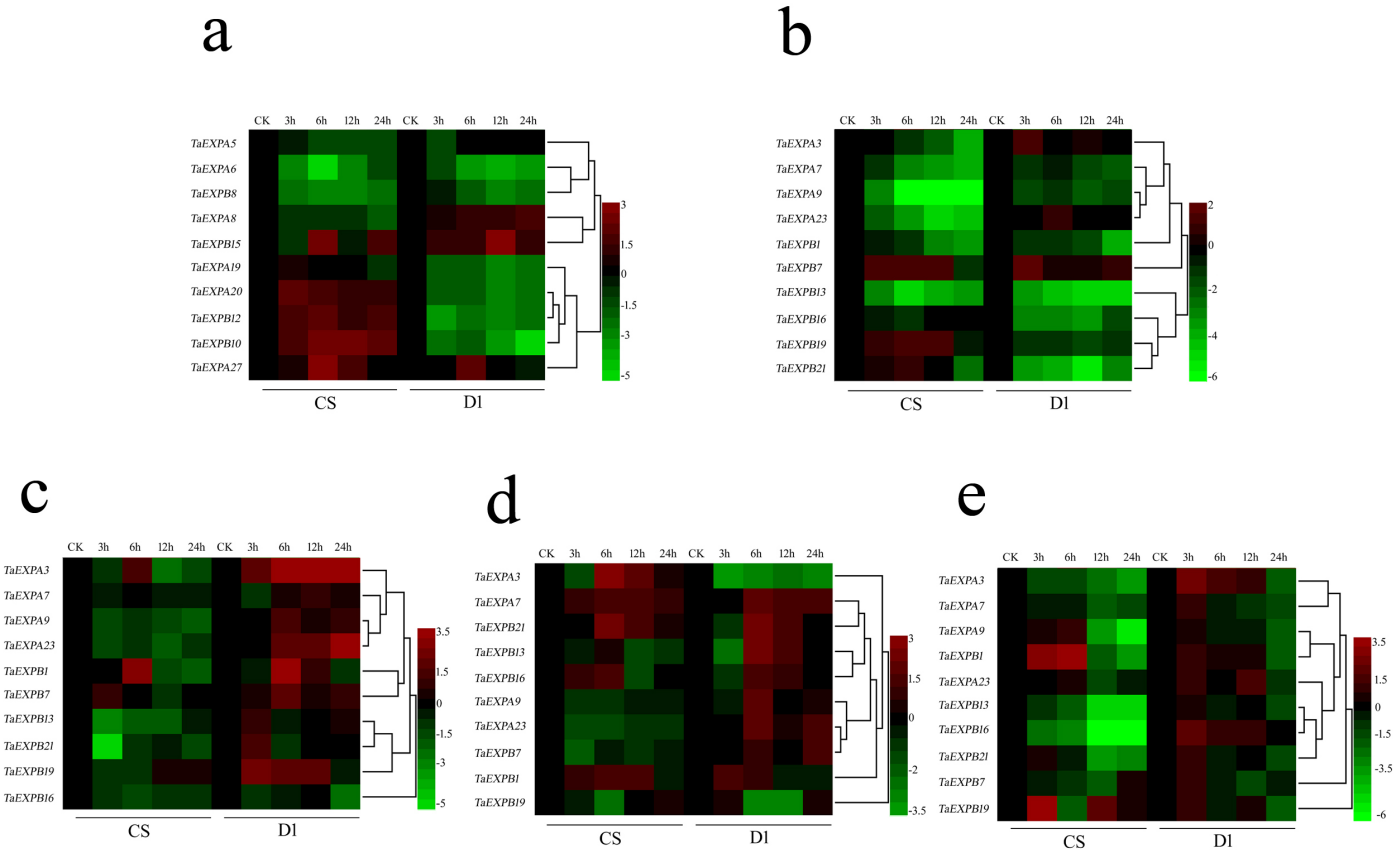
Differential expansin expression in CS and D1 during the three leaf stage at 4°C, compared against the expression at normal growth condition at the same time point. a–b. roots in CS and D1; c. tillering nodes in CS and D1; d. stem in CS and D1; e. leaves in CS and D1. Results were shown as average of three independent experiments.

We further selected 10–20 *TaEXP* genes to compare their expressions under low-temperature (4°C) treatment at different time points (from 0 to 24 h). Our results from each independent experiment had a high consistency. So the following results (heat maps) were shown as the average of three independent experiments. Many of the *TaEXP* genes showed significant differences in expression between CS and D1, especially after 24 h of low temperature treatment (Fig 6a–6e). In root (Fig 6a and 6b), the expression levels of most of the *TaEXP* genes decreased as the time at 4°C increased from 3 to 24 h. Many of the *TaEXP* genes were found differentially expressed. The expressions of *TaEXPA20*, *TaEXPB10*, and *TaEXPB12* increased in CS but decreased in D1 at 4°C, while the expression of *TaEXPA8* decreased in CS but increased in D1. The expressions of *TaEXPA5*, *TaEXPA6*, and *TaEXPB8* decreased in both CS and D1.

In tillering nodes (Fig 6c), overall, the expression of *TaEXP* decreased in CS, but increased in D1. Considering the phenotype of CS at 4°C, the decrease of *TaEXP* expression may be related to the collapse of the CS plants. In D1, the expressions of five genes increased during 3–6 h of 4°C treatment. A similar increase of expression of *TaEXPA9*, *TaEXPA23*, and *TaEXPB7* was also observed in both CS and D1 during the tillering stage, suggesting that these three genes may play important roles in tillering node development. In stem (Fig 6d), the expression of most of genes in CS decreased, while they increased in D1, suggesting that the decrease in CS may be involved in the collapse of the plants under low-temperature stress. Fig 6e shows the *TaEXP* expression changes in leaves. The expressions of most of the genes decreased in both CS and D1, but the decrease, which occurred mostly during 12–24 h of low-temperature treatment, was more significant in CS than in D1. The slower decrease of *TaEXP* expression in D1 may facilitate the low-temperature tolerance by giving the plants more time to adjust this type of environment change.

Overall, the differential expression was more prominent between CS and D1 in root-specific *TaEXP* genes. This could be one of the causes of the significant difference of root development between the two cultivars. The expression differences in leaves and nodes were less significant between CS and D1, suggesting the *TaEXP* genes may have similar functions in the development of leaves and nodes.

## Discussion

Expansins are a large family of proteins that play broad roles in plant development, from the emergence of root hairs to fruit softening. Recent studies have shown documented the presence of 36 expansin genes in *Arabidopsis*, 75 in soybean, 52 in tobacco, 38 in tomato, 39 in potato, 58 in rice, and 88 in maize [31]. Because of the complexity of the wheat genome, only 18 expansins have been identified so far [28]. In this study, we identified 128 expansin sequences in wheat using a genome-wide approach. These sequences belong to 45 different wheat expansins. Further study of these expansins will significantly enhance our understanding the physiological functions of wheat expansins.

It has been reported that the number of expansins in each of the four subfamilies of expansins differs significantly [31]. In most cases, the number of EXPAs are larger than that of EXPBs. Consistent with this report, we identified 26 *TaEXPAs* and 15 *TaEXPBs*. One interesting phenomenon is that the number of *TaEXPBs* is higher in monocots than in dicots [31]. One possible explanation for this is that *EXPAs* are more specific to xyloglucans, which are more abundant in the primary walls of non-graminaceous plants [56]. However, *EXPBs* are more specific to xylan [2], which is a minor component of the primary walls of dicots but abundant in the walls of grasses of the Poaceae [57]. Another difference of expansin genes in monocots and dicots was the proportion of introns extended genes, which was less than 15% in wheat (Fig 1c) and maize (monocotyledon), but more than 75% in dicots [31].

*Cis*-acting elements are short DNA sequences on the promoter regions. These elements are recognized and bonded by trans-acting factors that control gene expression. Having the same or similar *cis*-acting elements on different genes often suggests that these genes are being transcribed in a coordinated way. In this study, we found that stress-related *cis*-acting elements are abundant and common in many EXPs. For example, 10 to 14 TGACG/CGTCA-motifs were found in 17 EXPs, including ten EXPBs, six EXPAs and one EXLA (S2 Table). TGACG/CGTCA-motifs are involved in jasmonic acid methyl ester (MeJA) responses and play important roles in the response of plants to drought and low temperature stress. MeJA, a volatile organic compound, is used in plants for stress response and in many diverse developmental pathways such as root growth or flowering. It could accelerate the expression of *EgEXPA2* and *EgEXPA3* in petals [58]. Aside from *cis*-acting elements, gene expression of *TaEXPs* is also regulated by DNA methylation in plants [59]. A higher level of cytosine methylation was detected in the *TaEXPA1-B* promoter, making *TaEXPA1-B* silent and activating *TaEXPA1-A* and *D* [27]. However, these three homoeologous *TaEXPA1* genes showed a similar phenotype and function in transgenic *Arabidopsis* [29].

The development of wheat roots is essential to overwintering and regeneration; studies have shown that expansins play important roles in root development. In *Brachypodium* root, auxin influenced the up-regulated expression of 23 expansins [60]. In barley (*Hordeum vulgare*), expression of the *HvEXPB1* was found to be root specific. A lack of *HvEXPB1* expression led to the spontaneous generation of a root-hairless mutant barley [61]. *TaEXPB12* shares 97% similarity with *HvEXPB1* and may have similar functions. Our current results showed that *TaEXPB12* was expressed in root at a low level in wheat. The rice (*Oryza sativa*) expansin *OsEXPA17* was exclusively expressed in root hair cells and mutants of *OsEXPA17* had shorter root hairs [8]. Limited *AtEXPA7* expression also led to shorter root hair development in *Arabidopsis* [7]. This suggests that root hair development requires a synergistic action between EXPAs and EXPBs [37]. Moreover, *AtEXPA17* and *AtEXPA18* were shown to be involved in the formation of primordia in lateral root [62, 63]. In our current study, we showed that the cold-tolerant D1 wheat cultivar had longer and stronger roots, compared to the cold-sensitive CS cultivar.

Studies have shown that the major function of expansins is to change the strength and elasticity of cell walls by modifying the polysaccharide bonds in those walls. The activation of expansins loosens the cell wall, while the inhibition of expansin expression reduces the flexibility of cell walls (Cosgrove 2000a). Expansin is the only type of protein discovered to date that can change the elasticity of cell walls but not damage its viability, suggesting that expansin only breaks the hydrogen bonds of the cell wall polysaccharides and that this process is reversible [2, 3].

The differential expressions of many expansin genes between the CS and D1 cultivars suggest that expansins in D1 could help enhance its low-temperature tolerance by modifying the cell wall elasticity. Further studies will be required to understand which expansin genes are involved in this process.

Wheat and other plants have evolved multiple mechanisms to respond to environmental stress, such drought, high levels of salt in soil, and high/low temperatures. Understanding the molecular mechanisms may help us select stress-tolerant cultivars and improve crop yields. Our real-time qPCR analysis showed that many *TaEXPs* were differently expressed in the CS and D1 wheat cultivars. These differential expressions may cause differential development of different organs. For example, we found that the expression of *TaEXPB10* and *TaEXPB12* was much higher in CS than in D1 (Fig 6a). The differential expressions in root at 4°C (Fig 6a) might have partially caused of the collapse of CS under the low-temperature treatment. *TaEXPB12* has a 97% similarity to *HvEXPB1*, which has been shown to be directly related to the formation of root hair in barley [58]. We speculate that the D1 strain may enhance its low-temperature resistance by limiting its root hair development and therefore reducing the contact with soil. One limitation of our current study is the lack of comprehensive comparison of gene expressions of the same variant (CS or D1) between normal growth condition and cold treatment, due to the complexity of expansin expression at different time points under low temperature treatment. As a result, the differences of expansin gene expression between CS and D1 under cold treatment could potentially be contributed in part by their genetic background. Studies are underway in our laboratory to comprehensively compare the expansin gene expression under different growth conditions and in different tissues.

In conclusion, our genome-wide screening of wheat expansins identified more expansin genes than had been previously reported. Their differential expressions in different tissues suggest wheat expansins have complex functions. Our comparative expression study between cold-tolerant and cold-sensitive wheat cultivars revealed that expansins in wheat may be involved in a low-temperature response. Our study sheds new light on the functions of expansins in plant development and stress response.

## Supporting information

S1 Fig The of wheat expansin genes on chromosomes. Genes from IWGSC database were showed in physical map of wheat chromosomes.
(DOC)

S2 Fig Domain analysis of *TaEXP* proteins. DPBB_1 domains and Pollen_allerg_1 domains were contained in *TaEXP* proteins.
(DOC)

S3 Fig The schematic diagrams of motifs in *TaEXP* genes. There were 15 motifs in *TaEXP* genes.
(DOC)

S1 Table Primers used for PCR analysis. These primers were used in qPCR.
(DOC)

S2 Table The number of *cis*-acting elements identified in the *TaEXP* genes. The number of ABRE, ERE, GARE-motif, TGA-element, TCA-element, TGACG/CGTCA-motif and LTR was showed respectively.
(DOC)
