# Supplementary figures and images for: Genome-wide identification of wheat (Triticum aestivum) expansins and expansin expression analysis in cold-tolerant and cold-sensitive wheat cultivars
Source: PLoS One. 2018 Mar 29;13(3):e0195138. doi: 10.1371/journal.pone.0195138 (PMC5875846; doi:10.1371/journal.pone.0195138)

**
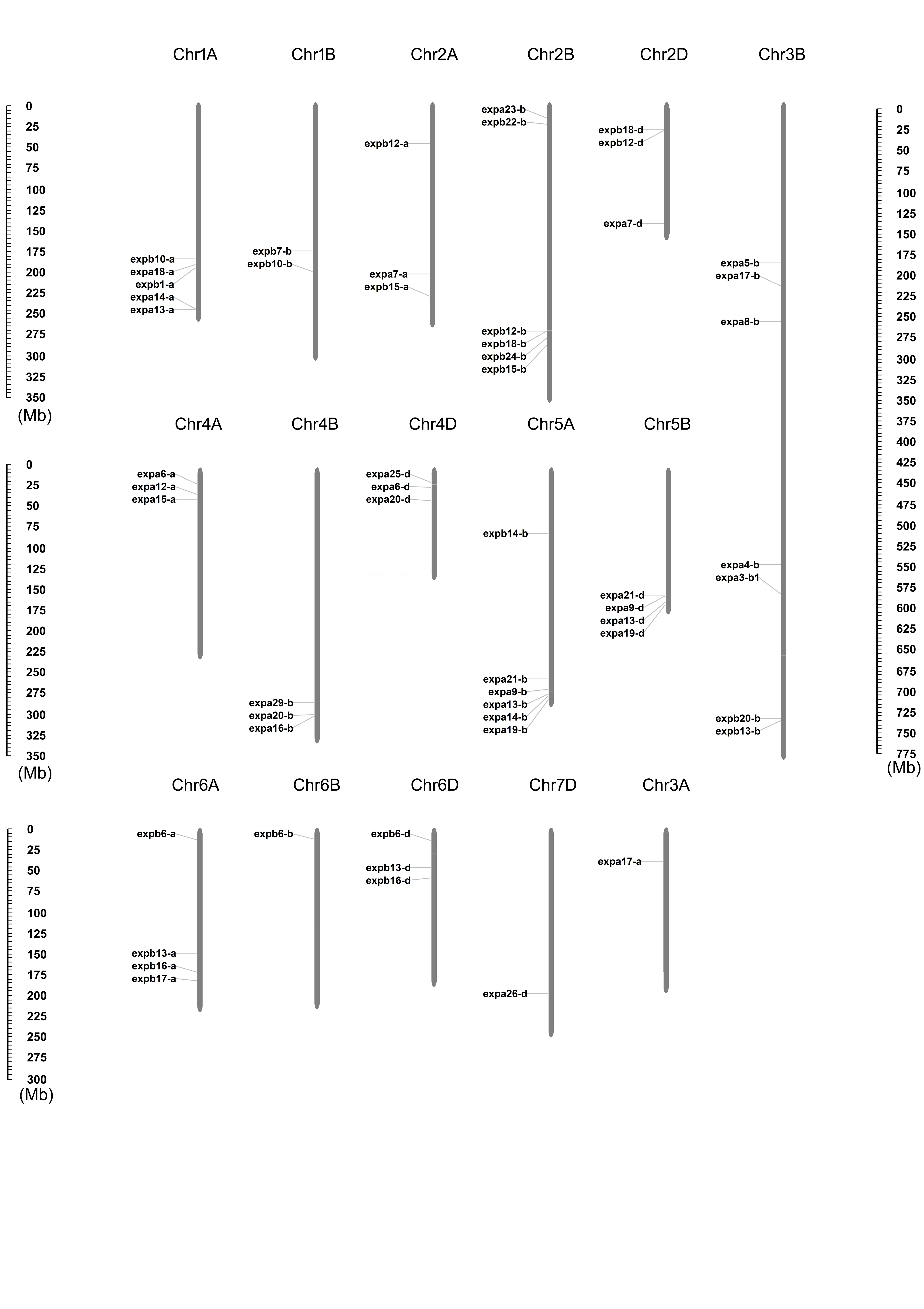
**

**Figure S1.** The physical map of wheat expansin genes on chromosomes.

Supplement: S1 Fig — Genes from IWGSC database were showed in physical map of wheat chromosomes. (DOC) [file pone.0195138.s001.doc]

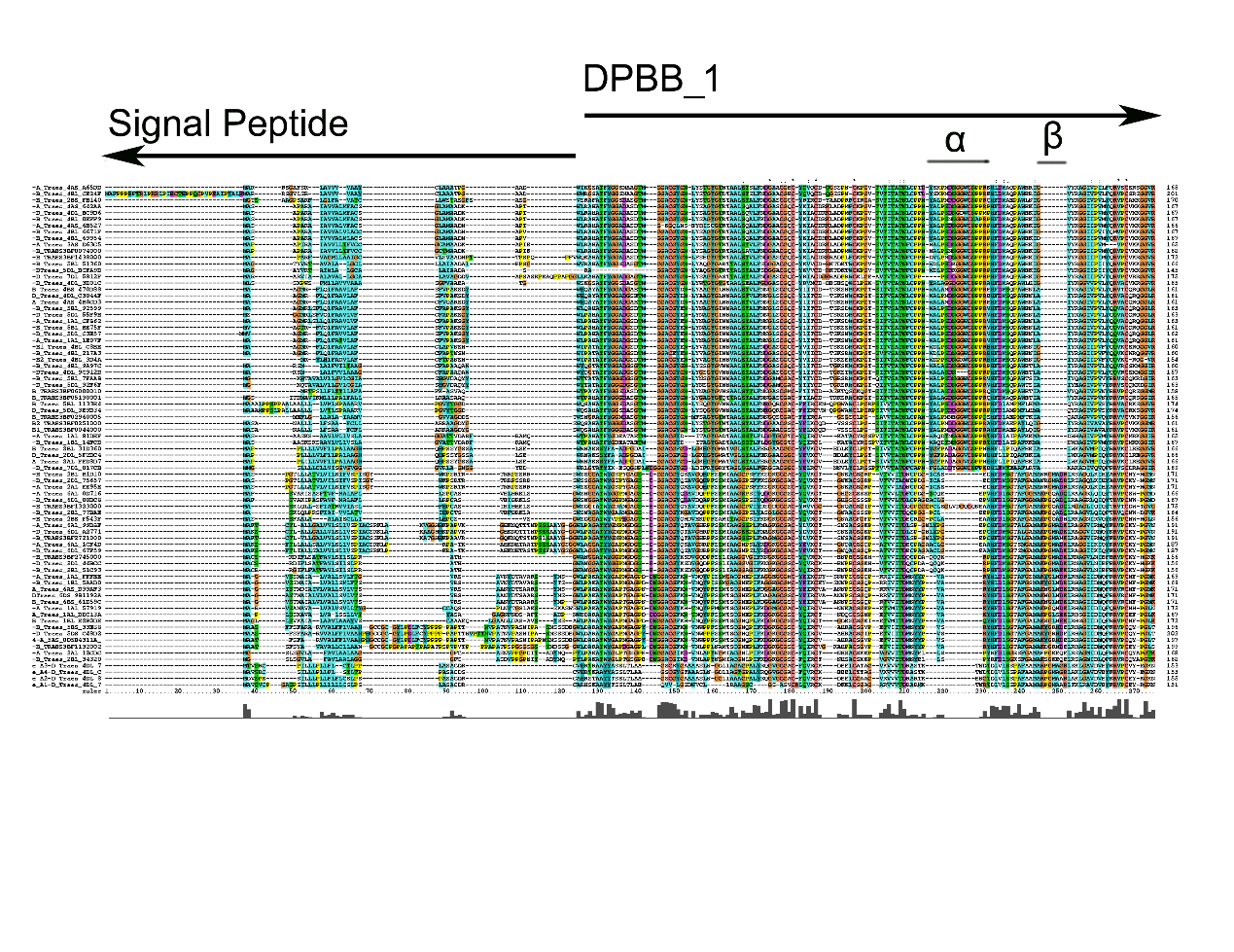


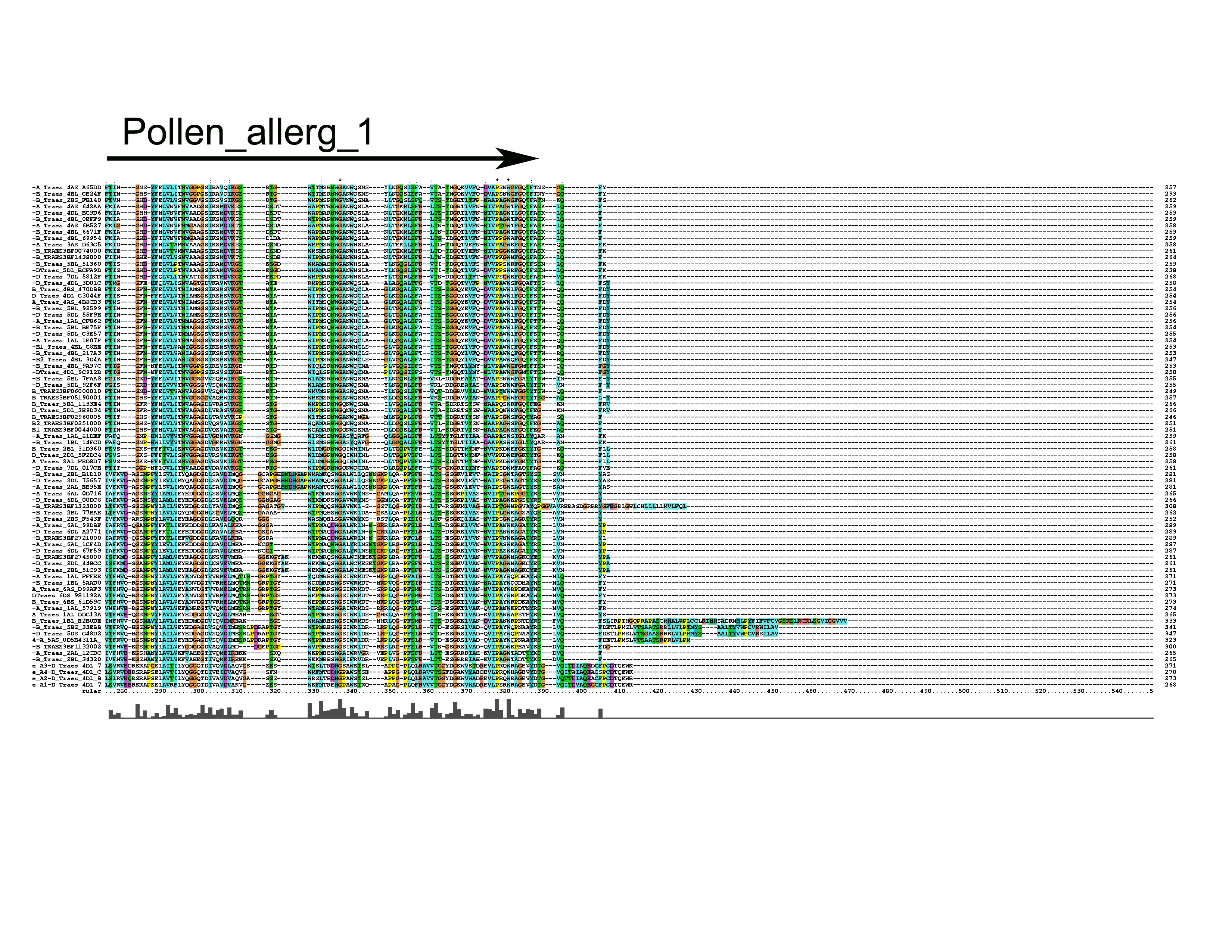


**Figure S2.** Domain analysis of *TaEXP* proteins.

Supplement: S2 Fig — DPBB_1 domains and Pollen_allerg_1 domains were contained in TaEXP proteins. (DOC) [file pone.0195138.s002.doc]
