# Supplementary material for: Genome-wide identification of wheat (Triticum aestivum) expansins and expansin expression analysis in cold-tolerant and cold-sensitive wheat cultivars
Source: PLoS One. 2018 Mar 29;13(3):e0195138. doi: 10.1371/journal.pone.0195138 (PMC5875846; doi:10.1371/journal.pone.0195138)
Supplement: S3 Fig — There were 15 motifs in TaEXP genes. (DOC) [file pone.0195138.s003.doc]

Motif 1


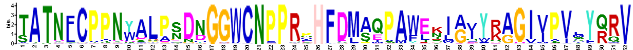


Motif 2


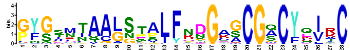


Motif 3


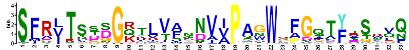


Motif 4


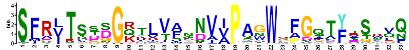


Motif 5


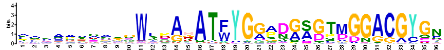


Motif 6


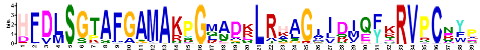


Motif 7


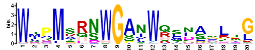


Motif 8


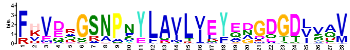


Motif 9


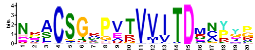


Motif 10


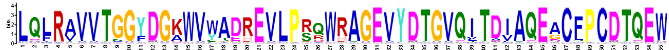


Motif 11


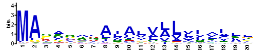


Motif 12


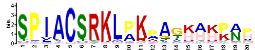


Motif 13


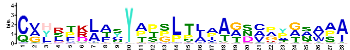


Motif 14


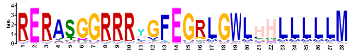


Motif 15


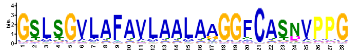


**Figure S3.** The schematic diagrams of motifs in *TaEXP* genes.
