# Supplementary material for: Genome-wide identification of wheat (Triticum aestivum) expansins and expansin expression analysis in cold-tolerant and cold-sensitive wheat cultivars
Source: PLoS One. 2018 Mar 29;13(3):e0195138. doi: 10.1371/journal.pone.0195138 (PMC5875846; doi:10.1371/journal.pone.0195138)
Supplement: S1 Table — These primers were used in qPCR. (DOC) [file pone.0195138.s004.doc]

**Table S1. Primers used for PCR analysis**

β-actin

5’TCATCCTGTGTTGCTGACTGAG

3’ACAGAACGGCCTGGATTGC

TaEXLA1

5’ACGACGGCAAGTGGGTGT

3’GCGACATCGGTGATCTGGA

TaEXLA2

5’AGAACCTCTCGCTGCGTGTG

3’ACCGCGACGATGTCAGTCTG

TaEXLA3

5’GGCCATCACGATCCTCTACC

3’GCGACATCGGTGATCTGGA

TaEXLA4

5’AGAGGAACTCTGCACTACCG

3’TCGTTCGTCTTCGTGCTG

TaEXPA3

5’GCCTACCTCCAGATCGGAATC

3’CGTTGCTCTGCCAGTTCTG

TaEXPA4

5’CTGACTGGATGGTAATGAGCAG

3’CCGTTGAGGTAGGCATTGC

TaEXPA5

5’CTACTTCGAGCTGGTGCTG

3’GGCTCATCTCCATCCAGTTGG

TaEXPA6

5’GCACGGTGCTGTTCAACAAC

3’GGCTTGCACATATTCGACTTGC

TaEXPA7

5’CGTCAAGTGCCTTCGTGAAG

3’ATTAGGACTGTGAAGAAGAAGCTC

TaEXPA8

5’ACCTCGCTGAGCCTATGTTC

3’GGTGATGGTGAACCGGATGC

TaEXPA9

5’AGAACTGGCAGTCCAACGC

3’TCGAAGGTCTGTCCGAAGC

TaEXPA12

5’GTGTGGATACGGCAACCTGTAC

3’TAGCACTGACCGCATGACG

TaEXPA13

5’ACGGCGCTGTTCAACAATG

3’CATGTCTGACCTGCTGGTGTC

TaEXPA14

5’TGGTTCTCGCCTTCTCCTTC

3’CAGGTGATGAGGTAGCACTGC

TaEXPA15

5’CGATGCCTCTGACACCATG

3’ATCTGTGTTGGTCAATCTGAATGAG

TaEXPA17

5’TATTGCGGTGGTGCTGCTC

3’GCCGAGTACAGGTTACCATACC

TaEXPA18

5’ACTGGCTGCTGGTGTACGTC

3’TGTAGGAGGTGAGCTTGAAGC

TaEXPA19

5’GACTACTTCGAGCTGGTGGTG

3’CGAGCCAGTTGGTGTTGGAG

TaEXPA20

5’GATCTCCTTCTCCGTCACCTC

3’TAGCCGAACTGCTGGTTGC

TaEXPA21

5’GCAGTGCTACAAGGTGGTGTG

3’AGAAGTTGGTGGCGGTGACG

TaEXPA22

5’TCTGCGACACCAGCAAGTC

3’ACGGTAGATGCCGATGTTCTC

TaEXPA23

5’GTCAGGTTCACCGTCAACGG

3’TGATGGACACCGACCGGATG

TaEXPA24

5’CAATCTTGTGCTTGTGAGCAATG

3’GAATCAGAGGTCTTGACATCCATG

TaEXPA25

5’GGCTCCACATCGGCATCTAC

3’AGCTGGAAGTGCTCGAACC

TaEXPA26

5’AACGGCCATGACTACTTCCAG

3’CGTCTTGATCGAGCCAATGG

TaEXPA27

5’GAGGACGATCACCATGTATAACG

3’CTACTCGACGAACTGCTTGC

TaEXPA28

5’GGCAGACGCTTGTCTTCAAC

3’AATCGCTCCGTTGGCAGTG

TaEXPA29

5’GCTCAGCTTCACCATCACCTC

3’ACGACATCCTGGAAGACAAGG

TaEXPB1

5’ACCTCATGGAGGCCAACTCC

3’ATGACCTTGTCGGCCACCAG

TaEXPB7

5’TACTCTCTGTCAAGGCCATCG

3’AGACATACGCGGCAGCAAG

TaEXPB8

5’GTTCTGCGGCCAACTACGAC

3’GGTACTGGTTCACGTTCTTGAAG

TaEXPB10

5’GGATCGTCATCACCGACATG

3’CACTGAGGTCGAGGTGGTAC

TaEXPB12

5’CTCCTCCAGTCGAACAACGG

3’AGGTGAGCCGGAACGAGAAC

TaEXPB13

5’GCCACGTATTACGGCAACC

3’CGATCATCGACGAGAACGG

TaEXPB14

5’CCAACTACCTGGCGATCCTG

3’CCATTATGTCCACCTGCGACAC

TaEXPB15

5’GCTCCTGCTTCCAGATCAGG

3’CGTAGTTCATGTCCGTGATGAC

TaEXPB16

5’TGGCATGAACATTGCCTTCAAG

3’TTGGAGTTGAGGCGGTACAG

TaEXPB17

5’GGACATAGCCTTCAAGGTGGAC

3’GAGCATGGCGAGGTAGTAGG

TaEXPB18

5’GTGGTGCATGGAGTCCAAGAC

3’GGTGAGCCGGAAGGAGAATG

TaEXPB19

5’CGTGAACTTCCACGTCGAG

3’CTCCATGAGGTCCATCTGCAC

TaEXPB20

5’ACCTTAGCGGCACTGCCTTC

3’CCTCTTGAACTGCATGTCGATG

TaEXPB21

5’TTCAGGTCCGGCAAGATGC

3’ACAGCAGGAGGTGACACAGC

TaEXPB22

5’AGCTGGCAACATTAGAGTCCAG

3’CTGCGAGGTAGTTCGGATTG

TaEXPB24

5’TCCAGCTCTGGCAAGAAGC

3’AATTGACCGCCGACTGGTAC
