# Supplementary material for: Genome-wide identification of wheat (Triticum aestivum) expansins and expansin expression analysis in cold-tolerant and cold-sensitive wheat cultivars
Source: PLoS One. 2018 Mar 29;13(3):e0195138. doi: 10.1371/journal.pone.0195138 (PMC5875846; doi:10.1371/journal.pone.0195138)
Supplement: S2 Table — The number of ABRE, ERE, GARE-motif, TGA-element, TCA-element, TGACG/CGTCA-motif and LTR was showed respectively. (DOC) [file pone.0195138.s005.doc]

**Table S2. The number of cis-acting elements identified in the *TaEXP* genes**

| **Gene Name** | **ABRE** | **ERE** | **GARE-motif** | **TGA-element** | **TCA-element** | **TGACG/CGTCA-motif** | **LTR** |
| --- | --- | --- | --- | --- | --- | --- | --- |
| EXPA3-A | 5 | 0 | 0 | 0 | 0 | 2 | 0 |
| EXPA3-B1 | 3 | 1 | 0 | 2 | 1 | 6 | 0 |
| EXPA3-B2 | 3 | 1 | 0 | 2 | 1 | 6 | 0 |
| EXPA3-D1 | 3 | 0 | 0 | 0 | 1 | 6 | 1 |
| EXPA3-D2 | 6 | 0 | 0 | 0 | 1 | 2 | 0 |
| EXPA4-A | 3 | 0 | 2 | 1 | 1 | 2 | 0 |
| EXPA4-B | 1 | 1 | 1 | 0 | 3 | 8 | 2 |
| EXPA4-D | 2 | 0 | 0 | 0 | 4 | 8 | 1 |
| EXPA5-A | 0 | 0 | 0 | 0 | 0 | 8 | 0 |
| EXPA5-B | 2 | 0 | 1 | 4 | 0 | 10 | 0 |
| EXPA5-D | 1 | 0 | 0 | 0 | 0 | 2 | 1 |
| EXPA6-A | 2 | 0 | 0 | 1 | 1 | 2 | 0 |
| EXPA6-B | 1 | 0 | 0 | 1 | 1 | 4 | 1 |
| EXPA6-D | 1 | 0 | 0 | 1 | 1 | 0 | 0 |
| EXPA7-A | 5 | 0 | 2 | 0 | 1 | 4 | 3 |
| EXPA7-B | 3 | 0 | 1 | 0 | 2 | 4 | 1 |
| EXPA7-D | 5 | 0 | 1 | 1 | 0 | 0 | 0 |
| EXPA8-A | 0 | 0 | 1 | 0 | 0 | 0 | 0 |
| EXPA8-B | 1 | 0 | 1 | 0 | 0 | 2 | 3 |
| EXPA8-D | 0 | 0 | 1 | 1 | 0 | 0 | 2 |
| EXPA9-A | 4 | 0 | 0 | 0 | 1 | 0 | 2 |
| EXPA9-B | 4 | 0 | 1 | 1 | 1 | 8 | 1 |
| EXPA9-D | 4 | 0 | 1 | 0 | 2 | 0 | 1 |
| EXPA12-A | 4 | 0 | 1 | 0 | 0 | 8 | 0 |
| EXPA12-B | 2 | 0 | 0 | 1 | 0 | 6 | 0 |
| EXPA12-D | 4 | 0 | 1 | 1 | 0 | 6 | 0 |
| EXPA13-A | 4 | 0 | 1 | 0 | 2 | 2 | 0 |
| EXPA13-B | 0 | 0 | 1 | 0 | 1 | 0 | 0 |
| EXPA13-D | 1 | 1 | 0 | 1 | 0 | 6 | 2 |
| EXPA14-A | 0 | 0 | 0 | 0 | 1 | 8 | 0 |
| EXPA14-B | 2 | 0 | 0 | 0 | 1 | 2 | 0 |
| EXPA14-D | 1 | 0 | 0 | 0 | 3 | 2 | 0 |
| EXPA15-A | 3 | 1 | 0 | 0 | 0 | 2 | 0 |
| EXPA15-B | 1 | 1 | 0 | 0 | 3 | 6 | 0 |
| EXPA15-D | 1 | 2 | 0 | 0 | 1 | 6 | 1 |
| EXPA16-A | 3 | 0 | 0 | 0 | 0 | 6 | 2 |
| EXPA16-B | 4 | 0 | 0 | 0 | 1 | 4 | 1 |
| EXPA16-D | 2 | 0 | 0 | 2 | 1 | 8 | 0 |
| EXPA17-A | 4 | 0 | 1 | 1 | 1 | 8 | 0 |
| EXPA17-B | 2 | 0 | 1 | 0 | 1 | 10 | 2 |
| EXPA17-D | 2 | 0 | 0 | 1 | 1 | 6 | 1 |
| EXPA18-A | 2 | 1 | 0 | 0 | 1 | 0 | 0 |
| EXPA18-B | 2 | 0 | 0 | 0 | 2 | 6 | 0 |
| EXPA18-D | 1 | 0 | 0 | 1 | 2 | 0 | 0 |
| EXPA19-A | 1 | 0 | 1 | 1 | 3 | 4 | 1 |
| EXPA19-B | 1 | 0 | 2 | 2 | 3 | 4 | 0 |
| EXPA19-D | 1 | 0 | 1 | 1 | 3 | 8 | 0 |
| EXPA20-A | 3 | 0 | 0 | 0 | 4 | 14 | 1 |
| EXPA20-B | 1 | 0 | 2 | 0 | 0 | 4 | 0 |
| EXPA20-D | 2 | 1 | 1 | 1 | 0 | 4 | 0 |
| EXPA21-A | 5 | 0 | 0 | 2 | 1 | 2 | 1 |
| EXPA21-B | 6 | 0 | 0 | 0 | 4 | 0 | 2 |
| EXPA21-D | 4 | 0 | 1 | 1 | 2 | 4 | 0 |
| EXPA22-A | 2 | 0 | 0 | 3 | 2 | 2 | 0 |
| EXPA22-B1 | 1 | 1 | 0 | 0 | 2 | 2 | 0 |
| EXPA22-B2 | 1 | 0 | 0 | 1 | 1 | 0 | 0 |
| EXPA22-D1 | 0 | 0 | 1 | 0 | 1 | 2 | 0 |
| EXPA22-D2 | 1 | 0 | 0 | 0 | 0 | 4 | 0 |
| EXPA23-A | 0 | 0 | 1 | 0 | 0 | 10 | 3 |
| EXPA23-B | 0 | 0 | 0 | 0 | 2 | 4 | 1 |
| EXPA23-D | 2 | 0 | 0 | 0 | 0 | 12 | 2 |
| EXPA24-A | 4 | 0 | 4 | 0 | 1 | 8 | 0 |
| EXPA24-B | 2 | 0 | 1 | 0 | 0 | 2 | 0 |
| EXPA24-D | 4 | 0 | 2 | 0 | 0 | 8 | 3 |
| EXPA25-B | 3 | 0 | 0 | 0 | 2 | 4 | 0 |
| EXPA25-D | 2 | 0 | 0 | 0 | 2 | 12 | 0 |
| EXPA26-D | 1 | 0 | 1 | 0 | 1 | 4 | 2 |
| EXPA27-A | 2 | 1 | 1 | 0 | 0 | 0 | 1 |
| EXPA27-B | 0 | 0 | 0 | 0 | 0 | 6 | 1 |
| EXPA27-D | 0 | 0 | 0 | 1 | 5 | 4 | 1 |
| EXPA28-B | 2 | 1 | 0 | 0 | 3 | 8 | 0 |
| EXPA29-B | 0 | 1 | 0 | 2 | 6 | 4 | 1 |
| EXPB1-A | 4 | 0 | 0 | 0 | 2 | 10 | 0 |
| EXPB1-B | 2 | 0 | 0 | 0 | 2 | 4 | 0 |
| EXPB1-D | 1 | 0 | 0 | 0 | 2 | 4 | 0 |
| EXPB7-A | 2 | 0 | 0 | 2 | 1 | 10 | 0 |
| EXPB7-B | 2 | 0 | 2 | 2 | 0 | 2 | 0 |
| EXPB7-D | 2 | 0 | 0 | 2 | 1 | 6 | 0 |
| EXPB8-A | 3 | 1 | 0 | 2 | 0 | 6 | 1 |
| EXPB8-B | 1 | 0 | 0 | 3 | 0 | 2 | 1 |
| EXPB8-D | 2 | 0 | 1 | 1 | 0 | 2 | 0 |
| EXPB10-A | 6 | 0 | 0 | 2 | 2 | 0 | 0 |
| EXPB10-B | 2 | 0 | 1 | 1 | 2 | 4 | 0 |
| EXPB10-D | 3 | 0 | 0 | 0 | 1 | 2 | 0 |
| EXPB12-A | 7 | 0 | 0 | 1 | 1 | 12 | 0 |
| EXPB12-B | 3 | 0 | 2 | 0 | 2 | 4 | 0 |
| EXPB12-D | 2 | 1 | 1 | 0 | 0 | 4 | 1 |
| EXPB13-A | 3 | 0 | 1 | 0 | 1 | 10 | 1 |
| EXPB13-B | 1 | 0 | 0 | 0 | 0 | 10 | 1 |
| EXPB13-D | 0 | 1 | 0 | 0 | 0 | 4 | 2 |
| EXPB14-A | 3 | 0 | 1 | 2 | 0 | 10 | 0 |
| EXPB14-B | 4 | 0 | 1 | 0 | 0 | 2 | 0 |
| EXPB14-D | 3 | 0 | 0 | 0 | 1 | 4 | 0 |
| EXPB15-A | 2 | 0 | 1 | 3 | 1 | 6 | 0 |
| EXPB15-B | 1 | 0 | 1 | 1 | 0 | 10 | 0 |
| EXPB15-D | 3 | 0 | 0 | 1 | 0 | 8 | 0 |
| EXPB16-A | 3 | 0 | 0 | 0 | 1 | 4 | 1 |
| EXPB16-B | 2 | 0 | 1 | 0 | 2 | 0 | 0 |
| EXPB16-D | 3 | 0 | 1 | 0 | 0 | 0 | 0 |
| EXPB17-A | 2 | 1 | 0 | 0 | 1 | 4 | 2 |
| EXPB17-D | 4 | 0 | 1 | 1 | 0 | 4 | 1 |
| EXPB18-A | 2 | 0 | 0 | 3 | 0 | 0 | 0 |
| EXPB18-B | 2 | 1 | 2 | 1 | 0 | 6 | 0 |
| EXPB18-D | 1 | 0 | 0 | 0 | 0 | 6 | 0 |
| EXPB19-A | 2 | 0 | 2 | 1 | 0 | 12 | 1 |
| EXPB19-B | 1 | 0 | 0 | 1 | 1 | 10 | 0 |
| EXPB19-D | 2 | 0 | 1 | 0 | 0 | 12 | 1 |
| EXPB20-B | 1 | 0 | 0 | 0 | 0 | 4 | 0 |
| EXPB20-D | 2 | 0 | 0 | 2 | 0 | 2 | 0 |
| EXPB21-A | 0 | 0 | 1 | 0 | 2 | 2 | 0 |
| EXPB21-B | 0 | 0 | 1 | 0 | 2 | 2 | 0 |
| EXPB21-D | 1 | 0 | 0 | 0 | 1 | 2 | 0 |
| EXPB22-A | 3 | 0 | 0 | 0 | 1 | 4 | 0 |
| EXPB22-B | 2 | 1 | 1 | 1 | 1 | 6 | 0 |
| EXPB22-D | 3 | 0 | 1 | 1 | 2 | 4 | 0 |
| EXPB24-A | 2 | 1 | 0 | 1 | 0 | 8 | 1 |
| EXPB24-B | 3 | 1 | 1 | 0 | 0 | 2 | 1 |
| EXPB24-D | 2 | 2 | 0 | 0 | 1 | 6 | 0 |
| EXLA1-A | 6 | 0 | 1 | 2 | 0 | 4 | 0 |
| EXLA1-B | 1 | 0 | 1 | 0 | 0 | 2 | 0 |
| EXLA1-D | 3 | 0 | 1 | 2 | 0 | 8 | 2 |
| EXLA2-A | 3 | 0 | 2 | 3 | 2 | 6 | 0 |
| EXLA2-B | 0 | 0 | 1 | 0 | 3 | 2 | 0 |
| EXLA2-D | 1 | 0 | 0 | 1 | 1 | 6 | 2 |
| EXLA3-A | 0 | 0 | 0 | 1 | 0 | 6 | 1 |
| EXLA3-B | 1 | 0 | 0 | 2 | 0 | 8 | 0 |
| EXLA3-D | 0 | 0 | 3 | 0 | 1 | 8 | 1 |
| EXLA4-D | 0 | 0 | 0 | 2 | 1 | 10 | 0 |
